# Developing and Integrating Digital Sources in an Accessible and Sustainable Online Platform for Adolescents and Young Adult Cancer Survivors: Collaborative Design Approach

**DOI:** 10.2196/60897

**Published:** 2025-07-11

**Authors:** Carla Vlooswijk, Sophia HE Sleeman, Jonas Pluis, Daphne Bakker, Lisanne de Groot, Eveliene Manten, Peter Heine, Olga Husson, Mies C van Eenbergen, Pieter Vandekerckhove

**Affiliations:** 1 Department of Research and Development Netherlands Comprehensive Cancer Organisation Utrecht The Netherlands; 2 Dutch AYA 'Young & Cancer' Care Network Utrecht The Netherlands; 3 Kanker.nl Foundation Amsterdam The Netherlands; 4 Department of Medical Oncology The Netherlands Cancer Institute Amsterdam The Netherlands; 5 Department of Surgical Oncology Erasmus University Medical Center Rotterdam The Netherlands; 6 Department of Public Health Erasmus MC Cancer Institute Erasmus University Medical Center Rotterdam The Netherlands; 7 Delft Centre for Entrepreneurship Delft University of Technology Delft The Netherlands

**Keywords:** adolescents and young adults with cancer, online platform, internet, age-specific, collaborative design, peer support, patient involvement, design, teens, youth, development, quantitative questionnaire, patient experiences

## Abstract

**Background:**

Digital support for adolescent and young adult cancer survivors is fragmented and results in lacking a reliable overview of support services available to them. Collaborative design promises to integrate perspectives of diverse stakeholders and could help to develop an online platform, which increases access and has a long-term perspective. However, it has not yet been explored how collaborative design can be used more strategically to develop an online platform with these aims.

**Objective:**

This study aimed to explore how a collaborative design approach could be applied to develop an online platform for adolescent and young adult cancer survivors, focusing on three key objectives: integrating existing resources, improving accessibility, and ensuring long-term financial sustainability.

**Methods:**

In this action research study, we reflect on a collaborative design process to develop an online platform for adolescent and young adult cancer survivors. A quantitative questionnaire was sent out to adolescent and young adult cancer survivors and health care professionals. Stakeholders were actively engaged in stakeholder consensus meetings, and project management was carried out through monthly design meetings to facilitate coordination and decision-making of the development of the platform. Afterwards, a focus group was conducted among the project group to evaluate the collaborative design approach, analyzed using an inductive thematic approach.

**Results:**

Through the collaborative design approach, several Dutch organizations collaborated to develop, enhance, and combine online services for adolescent and young adult cancer survivors and their relatives. A dedicated online “young and cancer” platform for adolescent and young adult cancer survivors was developed, which integrates different types of information tools and supportive interactive elements from different sources. The integration of different resources into one platform improves the access and user experience of adolescent and young adult cancer survivors when it comes to online support. Through the reflection about the collaborative process, three themes were identified: (1) value of stakeholder participation; (2) conditions for working with adolescents and young adults with lived experience; and (3) collaboration between adolescents and young adults with lived experience and professionals from different backgrounds and organizations.

**Conclusions:**

A collaborative design approach can be used to efficiently develop an online platform for adolescent and young adult cancer survivors. The collaboration among professionals, including online developers, researchers, and adolescents and young adults with lived experience facilitated a direct translation of insights into the platform, while the support of the national cancer platform provides long-term sustainability. This study highlights the importance of strategic stakeholder selection and the intense involvement of stakeholders through a collaborative design approach.

## Introduction

Increasingly, the internet and digital technologies hold a great promise to provide additional care and support for patients with and survivors of cancer [1]. A growing number of digital interventions have been developed and designed for patients with cancer living with and beyond a cancer diagnosis [2-7]. These emerging technologies aim to support, educate, and empower cancer patients and survivors to manage their own health through the use of information platforms, online support groups, and self-monitoring applications [8]. Despite the careful development of these digital solutions, significant challenges remain regarding their uptake, accessibility, and reach [9], which hinders their ability to make a long-lasting impact [5]. These solutions are often developed as stand-alone solutions without a long-term strategy or appropriate integration into the existing architecture and without continuous funding consideration.

Support for adolescents and young adults with cancer is particularly underdeveloped. adolescent and young adult cancer survivors are defined as individuals diagnosed with cancer between the ages of 18 and 39 years [10,11]. A cancer diagnosis at such a young age has a huge impact and causes physical, psychological, and social challenges that differ significantly from those of older adults and children [12]. Young cancer survivors struggle with various- and often age-related questions in several areas, such as fertility, family, children, employment, and establishing (romantic) relationships [13]. Zebrack et al [14] showed that adolescent and young adult cancer survivors, especially those treated in adult clinical settings, reported unmet needs in regard to age-appropriate websites. Given that 96% of the Dutch adolescents and young adults in the age of 25-35 years use the internet in their daily life [15], this young group appears to be a suitable population for the use of these online technologies. Therefore, there is a potential to provide age-specific information to adolescent and young adult cancer survivors in a digital manner.

In the Netherlands, disease-related, age-specific information and online services are scattered and could be found on several websites of different organizations. Firstly, the Dutch adolescent and young adult “Young and Cancer” Care Network initiated a public web page with age-specific topics and a secured online adolescent and young adult4 community in 2012. In this adolescent and young adult community, adolescent and young adult cancer survivors could express feelings and exchange information, which has been found helpful in coping with cancer [16-18]. This informative landing page and community have been the starting point for further development and implementation of digital adolescent and young adult services. To access live peer-to-peer meetings or events, adolescent and young adult cancer survivors have to surf to other websites, such as the patient organization for young patients with cancers (Stichting Jongeren en Kanker [SJK]) [19] or “walk-in houses” for everyone who gets impacted by cancer [20]. Another example is the applications developed to empower and support adolescent and young adult cancer survivors, such as the adolescent and young adult Match app, aiming to support adolescent and young adult cancer survivors when it comes to keeping in contact with their relatives after their cancer diagnosis [21]. The fragmentation of all these services results in adolescent and young adult cancer survivors lacking a reliable overview of support services available to them. Therefore, a solution would need to be financially sustainable, easily accessible, and integrate the existing sources.

An age-specific online platform for adolescent and young adult cancer survivors could meet those needs, reducing the complexity of navigating multiple sources and ensuring that content is relevant to their distinct needs. A dedicated platform for adolescent and young adult cancer survivors could bring together different types of information, tools, and interactive elements, elevating the experience and efficiency of information access and support. Therefore, the Dutch adolescent and young adult “Young & Cancer” Care Network and SJK set out to improve and enhance online services for adolescent and young adult cancer survivors in the Netherlands. The COMPRehensive health outcome and intervention research among patients with adolescent and young adult cancer (COMPRAYA) study provided funding and a stepping stone for developing an online “young and cancer” platform together with and for adolescent and young adult cancer survivors [22].

From a methodological point of view, it can be challenging to develop an online platform that is financially sustainable, accessible, and integrated in collaboration with different stakeholders. Collaborative design could be promising here as it is widely used and has been shown to improve commitment, support the development and implementation of digital health applications and provide more tailored solutions to users’ needs [23-26]. Collaborative design can involve stakeholders to various degrees in a collective creative process whereby stakeholders are considered partners in the design process [23]. For instance, this approach involves creative workshops whereby diverse stakeholders collaborate in groups [27], and it can also involve interviews and observations [28]. Yet, it has been noted that overlooking long-term perspectives could potentially reduce the success of the implementation process [23]. Considering certain potential problems upfront in the innovation process can help to avoid costly mistakes in the future [29]. In this case, collaborative design could be strategically used by considering how to develop an online platform with a triple aim: (1) to integrate existing sources, (2) to make them more accessible, and (3) to have a financially sustainable perspective. To this end, we aimed to explore how a collaborative design approach could be applied to develop an online support platform for adolescent and young adult cancer survivors, focusing on these 3 key objectives.

## Methods

### Overview

This study follows an action research methodology [30,31], whereby we want to learn about the use of collaborative design methods through the development of the “young and cancer” platform for adolescent and young adult cancer survivors. Action research is ideally suited for studying innovation processes and working towards sustainable transformation as one generates research knowledge through practical experience [30]. We used a combination of quantitative and qualitative research methods.

### Hosting the “Young and Cancer Platform”

The aim to develop the Dutch online “young and cancer” platform stems from a broader research project called COMPRAYA [22]. The aim of the platform was to provide age-specific information, support, and potential digital interventions for adolescent and young adult cancer survivors. One of the conditions was to combine existing information and services and create new age-tailored services in a sustainable online place, preferably integrated into an existing organization or platform, such as Kanker.nl [7]. Within the collaborative design approach, the stakeholders were involved in the strategic choice for future-proof embedding in an already existing platform. In this way, we tried to avoid later problems with implementation, embedding, and financial sustainability. The Kanker.nl platform was chosen as the most suitable place to integrate the adolescent and young adult platform into. Kanker.nl is a centrally initiated Dutch online platform (on average 1.077.313 visits per mo, all ages) and structurally funded by the Dutch Cancer Society (Koningin Wilhelmina Fonds [KWF]). It is dedicated to providing information, support, and resources for patients with cancer and cancer survivors in general [7].

### Collaborative Design Approach

A collaborative design approach was a fundamental principle guiding the development of this online platform: nothing about adolescents and young adults, without adolescents and young adults [23]. Throughout the entire process, adolescent and young adult with lived experiences were at the forefront, from concept designing to prioritizing themes and decision-making.

The involvement matrix was used to show the roles of various stakeholders (adolescent and young adult cancer survivors, relatives, and health care professionals [HCPs]) throughout the developmental stages of diverse elements of the platform [32]. The involvement matrix is developed to help involve patient experts in research projects. It can help to give the collaboration of patient experts a central place in research, improve the collaboration, and report on collaboration in a systematic and clear manner. The roles are listener, co-thinker, advisor (gives [un]solicited advice), partner (works as an equal partner), and decision maker (takes initiative, [final] decision). A listener received information and updates, such as attending project meetings and being shown the information and tools that have been developed and will be available on the platform. A co-thinker is asked to give their opinion on already developed materials, for example, by testing online questionnaires and providing feedback on draft versions of tools and information for adolescent and young adult cancer survivors. An advisor is given (un)solicited advice in early stages, such as providing advice on themes and topics that need to be included and is given advice about prioritization of the implementation of the tools. A partner works as an equal partner, for example, writing materials or being a podcast host. A decision maker takes initiative and is in charge of final decisions.

**Figure 1:**
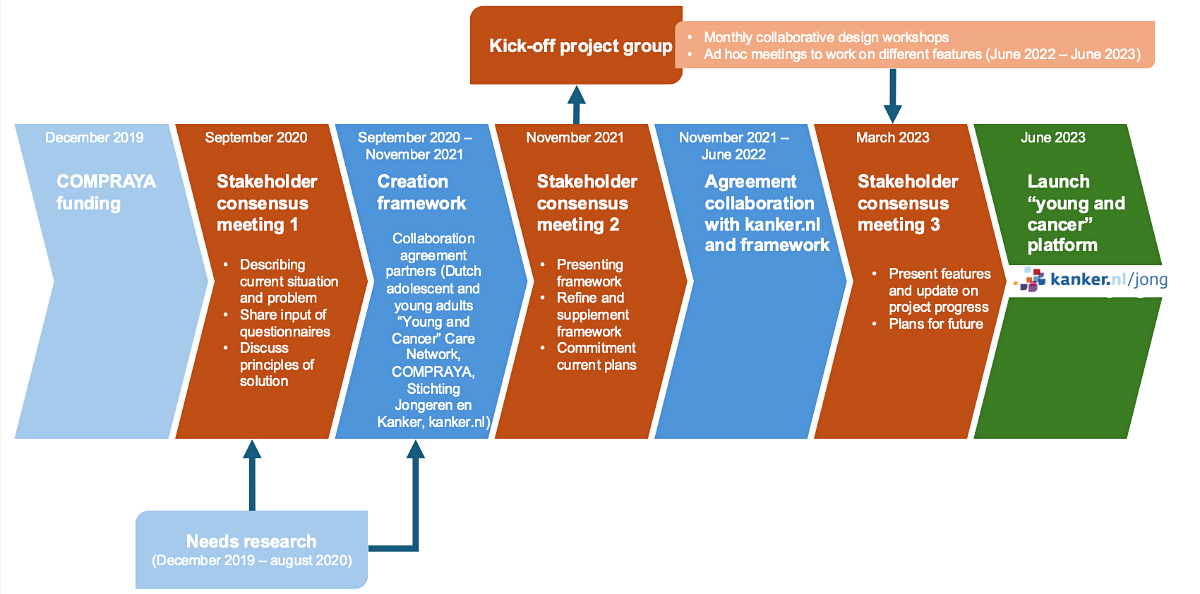
A graphical overview of the timelines of the collaborative design approach for the online “young and cancer” platform, showing the involvement of stakeholders from December 2019 to the platform’s launch in June 2023.

The collaborative design activities are shown in Figure 1. This process consisted of a needs research using quantitative questionnaires stakeholders were actively engaged in 3 stakeholder consensus meetings, a review group provided feedback ad hoc on developed content, and project management was carried out through collaborative design meetings to facilitate coordination and decision-making. These activities are further detailed below.

### Needs Research

Results of 2 conducted questionnaires were analyzed to understand the online wishes and needs among adolescent and young adult cancer survivors and the perspective of HCPs on this matter [33]. adolescent and young adult cancer survivors were invited via an online community, newsletters of the Nederlandse Federatie van Kankerpatiëntenorganisaties (NFK; Dutch Federation of Cancer Patient Organizations) and social media platforms and participants of the health-related quality of life and late effects among SURVivors of cancer in Adolescence and Young Adulthood (SURVAYA) study [34]. HCPs were recruited via a mailing from the Dutch adolescent and young adult “Young & Cancer” Care Network. These HCPs either already provided some form of integral adolescent and young adult care or were willing to do so [35].

### Collaborative Design Meetings

A project group coordinated the development of the “young and cancer” platform by taking part in monthly collaborative design meetings, once per 1 or 2 months, mostly online via Microsoft Teams. The project group consisted of adolescents and young adults with lived experience, a project leader, a project coordinator, a medical editor, and a business development manager. Within the monthly collaborative design meetings, the “young and cancer” platform was given shape through open discussion, exploring options, and taking into account principles of collaborative design, for example, equality [23]. In these meetings, regular evaluations of the approach, workload, and time investment were also conducted, with particular attention to the adolescents and young adults with lived experience who participated outside of their professional roles. This process was essential for ensuring continued alignment within the project group and to make sure that everyone was satisfied with the chosen approach and collaboration.

### Stakeholder Participation

Overall, 3 stakeholder consensus meetings were organized with people with 8 different backgrounds (medical specialists, medical social workers, nurse practitioners, board members of patient organizations, adolescent and young adult cancer survivors, relatives of adolescent and young adult cancer survivors, and online specialists). These 3 online meetings, each lasting 90 minutes, were conducted via Microsoft Teams and organized at 3 points during the different phases of the platform development (Figure 1). The aim of these meetings was to inform relevant stakeholders and act as an advisory board and provide ideas and suggestions. In addition, the meetings were used to build consensus and create support for the “young and cancer” platform among relevant stakeholders who play an important role in referring adolescents and young adults to this online platform.

### Review Group

An open call was extended to all adolescents and young adults and their relatives who were interested in providing feedback on the project group’s output and written materials from the perspective of their lived experiences.

In total, 20 adolescents and young adults and relatives were recruited through various recruitment places (eg, consultation room and social media). Reviewers from HCPs’ perspective were recruited via the stakeholder consensus meetings and asked to critically review the written materials for inaccuracies and supplement them with practical experiences. Researchers within the adolescent and young adult cancer field were asked to provide their input and feedback based on their research perspectives.

### Evaluation of the Collaborative Design Process

A focus group with the project group was conducted online via Microsoft Teams on April 12, 2023, for approximately 90 minutes. The focus group was done in accordance with the COREQ (Consolidated Criteria for Reporting Qualitative Research) guidelines for qualitative studies [36] (Multimedia Appendix 1). All project members of the project group were invited to participate by the project leader via email. Subsequently, the participants were contacted by the researcher and informed of the purpose of the focus group. All participants provided written informed consent to participate in the focus group. Before participation, participants self-reported sociodemographic and, if applicable, clinical information. The facilitator (CV), an independent trained female researcher, guided the focus group following a semistructured topic guide (Textbox 1).

**The topic guide of the focus group for evaluating the ‘collaborative design approach within the project group dedicated to developing the online “young and cancer” platform.**

**Topic guide:**

- Introduction
  - How is the division of roles and tasks within the project group?
  - What is your role in this project?
  - Why are you participating in this project?
  - When would you not have participated?
  - What is decisive? Time, money, people, and appreciation?
  - What is going well and what could be improved?
- Cooperation project group
  - Do you feel that you have influence on the project?
  - Is this (in)sufficient?
  - What is the added value of working in this project group?
  - Would you like to do this more often?
  - What helps you to do this?
- Online services for adolescents and young adults
  - When did the project succeed for you?
  - Which elements and issues are important to you in the final product (online platform for adolescents and young adults)?
  - What are the limiting or impeding factors?
  - Do you have tips or recommendations for others in similar projects?

### Data Analysis

Quantitative analyses were performed using SAS (SAS Institute) software. Descriptive statistics included frequencies, means, SDs, and percentages. The focus group was recorded in Microsoft Teams, transcribed verbatim, and analyzed via an inductive thematic approach described by Braun and Clarke [37] using NVivo (Lumivero). Initially, the focus group was reviewed by the researcher, and all the information describing the evaluation of the collaborative process was highlighted (open coding). Subsequently, these codes were discussed and agreed upon with SS.

### Ethics Approval

This project was approved by the Research Ethics Review Committee of the Tilburg School of Humanities and Digital Sciences (internal code: REDC 2019.104). Informed consent was obtained from all adolescent and young adult cancer survivors who participated in the questionnaire study and participants in the focus group. The adolescents and young adults with lived experience in our project were compensated with gift vouchers with approximately €50 (US $58.58) value per meeting.

## Results

### Needs Research

More than half (373, 63%) of the adolescent and young adult cancer survivors indicated that they wanted to find age-specific information and digital tools from various organizations converged into one website [33]. adolescent and young adult cancer survivors (43%) indicated that the priority should be on age-specific information on a website specifically designed for adolescent and young adult cancer survivors. Online peer contact was used by 18.4% of the adolescent and young adult cancer survivors. They mainly posted or responded on different platforms, such as Amazon Breast Cancer Support Group, patient organizations, their own websites, Facebook (Meta), Kanker.nl, Instagram (Meta), WhatsApp (Meta), and the adolescent and young adult community. More adolescent and young adult cancer survivors (38.3%) prefer to passively participate by reading online forums, blogs, discussion platforms, or patient communities. However, 6.4% could not find a suitable website and 12.6% prefer a website where mostly younger people with cancer gather. Only a small number (6.9%) of adolescent and young adult cancer survivors posed online questions to HCPs via, for instance, a secured online environment of the hospital or general practitioner, email, or WhatsApp. While 41.4% of adolescent and young adult cancer survivors indicated that they would like to ask questions of their HCP online, but they have never done this yet. More extended analyses based on these questionnaires to assess the quantity of cancer-related internet use, content searched for, perceived impact of cancer-related internet use, and eHealth needs among adolescent and young adult cancer survivors are published elsewhere [33,38]. These results were also compared with the perceptions of HCPs [38].

### Stakeholder Participation

#### Stakeholder Consensus Meeting 1

The project group presented the basic principles and scenarios for the suggested “young and cancer” platform and sought input from all stakeholders. Different scenarios were presented, such as the improvement or enrichment of websites of SJK and the Dutch adolescent and young adult “Young & Cancer” Care Network, where adolescent and young adult cancer survivors are familiar with finding this information *(*Multimedia Appendix 2), Other scenarios were using the existing platform of Kanker.nl. One of the scenarios even described building a completely new platform. The presented scenarios varying in terms of the amount of investment and complexity are required. Scenario 1 was “do nothing” and scenario 7 was building an entirely new, stand-alone platform, which required significant investment, short-term as well as long-term, and was more complex. A new channel integrated within an existing platform, Kanker.nl (a combination of scenarios 5 and 6), seemed to be the most reliable and optimal solution. This allows for the use of the infrastructure, maintenance, management, and overall development of the platform, while simultaneously creating a distinct look and feel for adolescent and young adult cancer survivors and presenting age-specific content. It could integrate existing sources, making the “young and cancer” platform more accessible and having a financially sustainable perspective. The existing features on Kanker.nl can be used and, where necessary, tailored for adolescent and young adult cancer survivors. A potential limitation of integrating the “young and cancer” platform into Kanker.nl is the existing principles and possible restrictions of the current platform, such as the available modals and infrastructure of its content management system.

#### Stakeholder Consensus Meeting 2

In total, 4 personas were used to provide a clear and concise way of ensuring everyone has a shared understanding of how the platform and features should function (Multimedia Appendix 3)*.* These personas represent adolescent and young adult cancer survivors and their relatives, including different sexes (male and female), tumor types (Hodgkin lymphoma, lung cancer, leukemia, and breast cancer), different ages (eg, 21, 23, 30, and 36 y), different times since diagnosis (currently under treatment, diagnosed 2 y ago), and varying needs on the platform (such as reading blogs, connecting with peers, accessing information files, using health applications).

The stakeholders highlighted several key features they wanted from the new platform, including Google search results linking to the “young and cancer” platform, dedicated promotional materials, an online calendar of available peer activities, the ability to join secure online support groups, the option to indicate openness to contact with other peers, and the ability to search personal profiles and reach out to suitable peers.

#### Stakeholder Consensus Meeting 3

The project group provided an update on the project’s progress and actively sought input and feedback from all stakeholders to gain a deeper understanding of their perspectives. They focused on refining, sharpening, and clarifying the plans to strengthen commitment to the current direction. To gain the most traffic to the new platform, the launch of the “young and cancer” platform was presented on June 15 June, 2023, during the SPACE4AYA congress for adolescent and young adult cancer survivors and their HCPs. Agreements on ongoing collaboration among stakeholders, COMPRAYA, SJK, Dutch “Young and Cancer” Care Network, and Kanker.nl ensure integration and further development of the “young and cancer” services in the future within the sustainable platform Kanker.nl.

### Development of the Online “Young and Cancer” Platform

Various features were developed within the new “young and cancer” platform. An overview of these features can be found in Table 1. The primary target group of the platform is individuals with cancer who have received a cancer diagnosis between the ages of 18 and 39 years (adolescents and young adults). The secondary target group are relatives of adolescents and young adults and Young Adult Childhood Cancer Survivors, those diagnosed with cancer before the age of 18 years, who are now between 16 and 39 years of age and can struggle with similar age-specific problems as adolescent and young adult cancer survivors. The framework, written by the project group and validated by the stakeholders, formulated the following concept: create a future-proof environment with reliable up-to-date information, the possibility to contact peers and access to eHealth applications. The following conditions were formulated: reuse of existing content with the possibility of adding age-specific information, reuse of existing features with the possibility to tailoring them to adolescents and young adults, ensuring a future-proof platform, continuation of maintenance when COMPRAYA funding ends, using results of scientific research from COMPRAYA and making the developed services for adolescents and young adults also available to other target groups if appropriate, for example, people with rare cancer.

**Table 1:** The stakeholder involvement in the development process for the various features of the online “young and cancer” platform is illustrated using the involvement matrix.

Features online platform			Involvement matrix [32]								
Roles			Listener		Co-thinker		Advisor		Partner		Decision-maker
Design landing page			—^a^		—		—		1^b^		1
Profile modifications for adolescent and young adult cancer survivors					1		—		—		—
**Age-specific information for adolescent and young adult cancer survivors and relatives**											
	Written information dossiers	—		2^c^, 3^d^,4^e^		—		1		—	
	Event calendar	1		—		—		1		—	
	Different types of content, for example, photography, visuals, ambassadors’ stories, podcasts, and videos	—		4				1		1	
**Opportunities for social interaction**											
	Blogs	1		—		1		—		—	
	Online support groups for adolescent and young adult cancer survivors and their relatives	—		4		1		—		—	
	Peer finder	—		1,2		2		1		—	
	Health care professional on demand	—		4		1		3		—	
	Guide for reliable information and support	1		—		1		—		—	
	Library for resources and a specific app store	—		—		1		—		—	

^a^Not Applicable.

^b^1: Project group.

^c^2: adolescent and young adult with lived experience and relative’s reviewers.

^d^3: health care providers and research reviewers.

^e^4: Stakeholder consensus meetings.

### Landing Page

The existing infrastructure of Kanker.nl was used to create a “landing page” for adolescents and young adults with an age-specific look-and-feel (including photography of young people) and offers the flexibility to vary in terms of design, navigation items, and content [39]. The users of the landing page can navigate to different age-specific features presented in their own unique tone of voice. The aim was to create an environment where adolescents and young adults feel seen and acknowledged, and to create an environment where adolescent and young adult cancer survivors could identify themselves with.

### Age-Specific Information for Adolescents and Young Adult Cancer Survivors

Age-specific content has been created, resulting in information dossiers for adolescent and young adult cancer survivors: disease and complaints, treatment and care, finance and insurance, fertility and pregnancy (Multimedia Appendix 4)*,* taking care of yourself, study and cancer, work and cancer, intimacy and sexuality, appearance, dealing with the people around you, emotions, and existential questions of life and palliative care. In addition, a separate information dossier for relatives, parents, partner, family, and friends was created. In addition to just text, both existing and newly developed interviews and podcasts related to adolescents’ and young adults’ cancer experiences were placed on the “young and cancer” platform. Furthermore, an events calendar with (online) activities for adolescent and young adult cancer survivors was placed. This calendar is linked to the calendar of SJK, which includes social gatherings, informational meetings, outdoor adventures, and other events that focus on connecting with peers.

### Opportunities for Social Interaction

**Figure 2:**
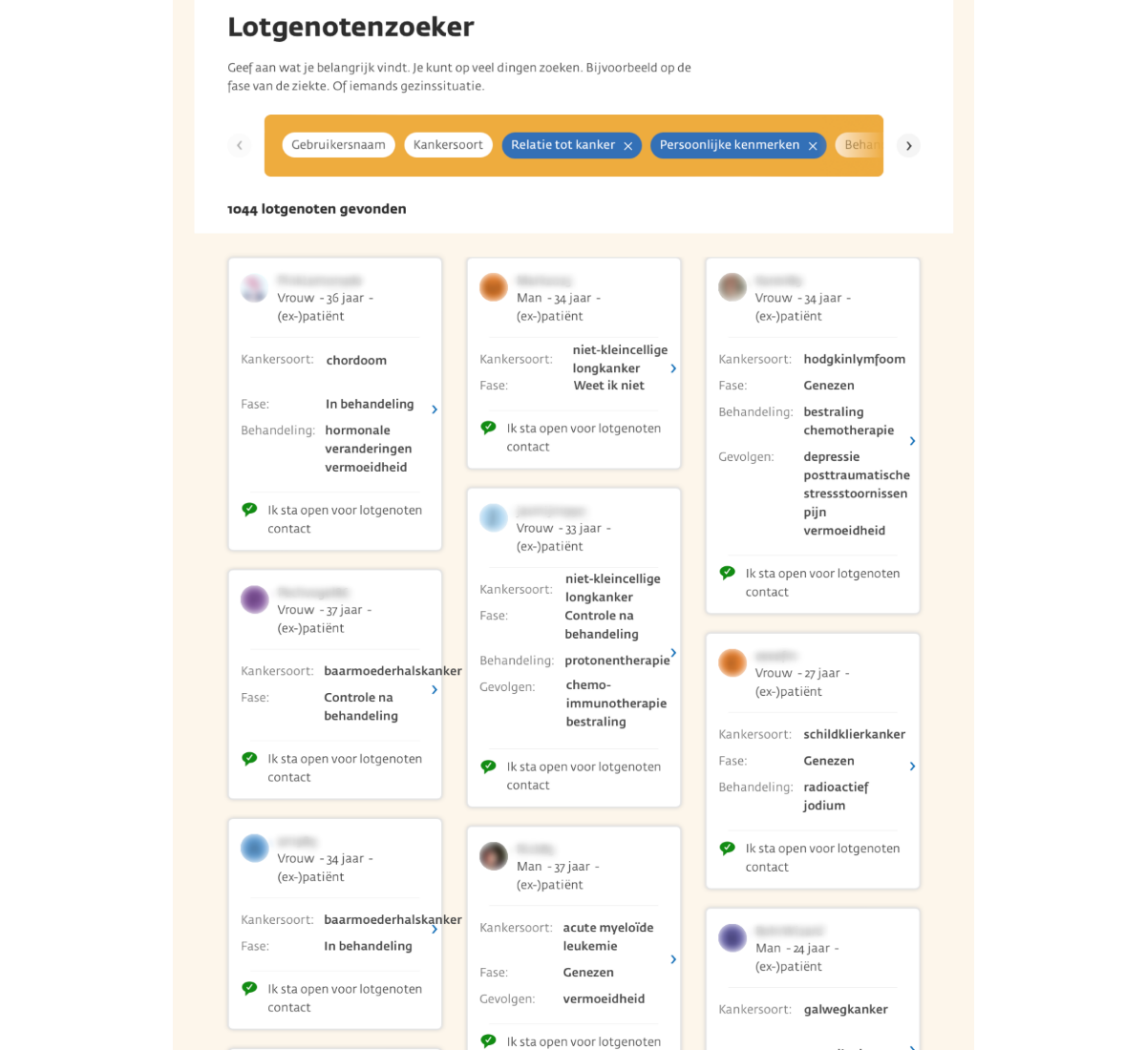
A screenshot of the “Peer finder”: a digital tool available on the online “young and cancer” platform to help cancer survivors find and connect with peers with cancer.

adolescent and young adult cancer survivors are able to read personal experiences of other (adolescent and young adult) cancer survivors and relatives in the blog section of Kanker.nl. They also have the opportunity to share their own experiences. A few blogs of adolescent and young adult cancer survivors and relatives have been highlighted, and an age-related filter will be put in place. The online support group, named “the young and cancer online support group,” exists on Kanker.nl. Thereby, an online support group especially for relatives of adolescent and young adult cancer survivors has been created, called “young and cancer-relatives.” Moderators (adolescents and young adults with lived experience and relatives of adolescent and young adult cancer survivors) have been appointed. The online support groups are open to registered members, allowing anyone with an account to read and participate. Visitors without an account can only view the conversation title unless the poster explicitly specifies that the content is fully public. To offer cancer survivors a safe and noncommittal way to get in touch with peers online, the “peer finder” was created. This is a tool in which patients with cancer can search for peers based on information from their personal profile, such as age, type of cancer, stage of the disease, treatment, and (late) effects (Figure 2). Cancer survivors and relatives can get in touch with people in a similar situation and directly send private messages in a secure environment.

### Health Care Professional on Demand

adolescent and young adult cancer survivors have the opportunity to (anonymously) pose online questions to HCPs with expertise in adolescent and young adult care. These questions and answers given by HCPs are publicly available to anyone. The HCP is an expert in the field, recognized by partner organizations, and preferably works in a multidisciplinary team within a network of colleagues/hospitals. Cooperation in this on-demand service from HCPs is on a voluntary (unpaid) basis.

### Guide for Reliable Information and Support

This guide is for patients with cancer and cancer survivors who are searching for support or additional care, for instance, HCPs (physiotherapists, social workers) with expertise in treating and supporting adolescents and young adults.

### Library for Resources and Specific App Store

The oncology app store consists of evidence-based and reliable applications. A couple of age-specific tools, including applications and decision aids can be found. These decision aids help adolescents and young adults in contemplating their treatment options and in preparing for discussion with HCPs.

### Evaluation of the Collaborative Design Process

All project members of the project group participated in the focus group. The demographic characteristics of the focus group (mean 44, SD 15 years; n=7) are presented in Table 2.

**Table 2:** The demographic characteristics of the project group participated in the focus group (n=7).

Characteristics				Values
**Sex, n (%)**				
		Male	5 (74)	
		Female	2 (29)	
**Current age (years)**				
	Range			22-66
	Mean (SD)			44 (15)
**Age at initial cancer diagnosis (years)**				
	Range			17-33
	Mean (SD)			27 (8)
**Living situation, n (%)**				
	Living alone			1 (14)
	Living with partner			4 (57)
	Living with children			2 (29)
**Highest achieved level of education, n (%)**				
	Secondary education or less			1 (14)
	Secondary vocational education			0 (0)
	Applied university			2 (29)
	University			4 (57)
**Employment status, n (%)**				
	Student			1 (14)
	Part-time work			3 (43)
	Full-time work			2 (29)
	Self-employed			1 (14)

The adolescents and young adults with lived experience in the project group considered the online platform for adolescent and young adult cancer survivors a success, as it effectively meets adolescent and young adult cancer survivors’ online needs. It should be used as a first starting point in the search for information, help adolescent and young adult cancer survivors to gain awareness and find recognition on what might hit them along the way, prevent them from getting lost or stuck on their search, or reach a dead-end. Furthermore, they aimed to lay the groundwork for the platform, ensuring it is not a one-time initiative but an ongoing resource that continuously monitors and updates important information and support for adolescent and young adult cancer survivors. A positive spin-off observed from the collaborative design approach was that the texts and topics developed were not only valuable for adolescent and young adult cancer survivors but also had relevance for older cancer survivors.

The evaluation of the collaborative design consisted of three themes, which are described below, and corresponding quotes are available in Multimedia Appendix 5.

### Value of Stakeholder Participation

The collaboration within a multistakeholder group avoided potential misunderstandings and missed opportunities during the development process. It enabled improvements in the information and services provided by the platform. The benefit of working with professionals from various organizations and expertise was the easy access to different expertise from different organizations and professionals. The understanding and inclusion of the perspectives of adolescents and young adults with lived experience was valuable for tailoring the online platform according to their needs and was important for validating the features.

### Conditions for Working With Adolescents and Young Adults With Lived Experience

Working with adolescents and young adults with lived experience required attention to several key factors. Not only did adolescent and young adult cancer survivors feel it was important to be heard, but they also valued recognition and the assurance that their contributions were taken seriously and used in the development of future services. adolescent and young adult cancer survivors wanted to be treated as equal partners within the project, to be involved from the beginning of the project and not just seen as a “checkbox” to be ticked off once they have participated. They preferred an open atmosphere where everyone was free to express their thoughts and feelings. Their main motivation to participate in the project group was that they wanted to improve information provision and care for future adolescent and young adult cancer survivors. However, a barrier to working as an adolescent and young adult with lived experience was the lack of financial compensation for their time and expertise, in contrast to other professionals. As a result, they often had to balance their work for the project group with other responsibilities, such as employment and child care. For this reason, the project leader felt responsible to not overburden them, by avoiding giving them additional time-consuming tasks, such as writing notes or producing materials. adolescent and young adult cancer survivors had to be capable of looking beyond their own situation and not basing their thoughts and actions solely on their own experiences. In addition, working in a project group with people with diverse (professional) backgrounds required attention to make jargon understandable for all involved.

### Collaboration Between Adolescents and Young Adults With Lived Experience and Professionals From Different Backgrounds and Organizations

The project group expressed that they really enjoyed working in this way. Working with different organizations and professionals provided the advantage of a financial and future-proof perspective while also incorporating a wide range of knowledge and skills into the project group. It was sometimes difficult for stakeholders to keep their own organization adequately informed and engaged in the process, as they were representing their organization in this external and interorganizational project. Throughout the project, efforts were made to explore ways to keep the wider organization informed and connected to the “young and cancer” project.

This interorganizational approach also required adherence to certain preconditions and established procedures over which there may have been little to no control. For instance, fundamentally, the design directions were limited by the web-hosting organization Kanker.nl; the solution could technically not be a serious game or a virtual reality experience, it was rather limited towards a static website. This resulted in having to compromise between the original framework and the given limitations of the existing platform. Expectation management and communication were crucial in this context, as the project team had specific wishes and requirements that needed to be addressed.

## Discussion

### Principal Findings

The “young and cancer” platform is integrated into the existing Kanker.nl platform using a collaborative design approach. The centralized location improves access to and overview of available digital support resources. The integration of these resources improves both the experience and efficiency for adolescent and young adult cancer survivors. The evaluation of the collaborative process identified three themes: values of stakeholder participation, conditions for working with adolescents and young adults with lived experience, and collaboration between adolescents and young adults with lived experience and professionals from different backgrounds and organizations.

In this study, we described how we involved different stakeholders, and especially adolescent and young adult with lived experiences in various different ways along the collaborative development process. This contributes to the lack of literature about how patients are involved in the development of digital interventions [40,41]. A systematic review from McCann et al [2], showed that only 14 studies reported involving young people in the development process, and 8 studies had expert input or included a steering group during the design phase. Patient involvement included focus groups [42,43], interviews [44,45], and questionnaires [46,47]. It also included usability testing [48], think-aloud testing [42], heuristic evaluation [42], and collaboration with research partners who participated in one-day meetings [49]. These meetings included plenary sessions, small group discussions, and individual assignments. However, there is limited evidence on how these patients were precisely involved in more detail. In our study, we explain different ways in how patients can be involved to develop different parts through a combination of questionnaires and the involvement of adolescents and young adults with lived experiences who were part of the project group and involved in decision-making. The involvement of adolescents and young adults with lived experience in research poses a number of challenges. Effective communication between researchers and patients is crucial; however, it can be problematic when technical jargon is used or when there is a lack of clear explanations about what is expected of adolescent and young adult cancer survivors. Furthermore, it is challenging to engage a diverse group of adolescents and young adults with lived experience, which can represent a wide range of perspectives. This population often has competing responsibilities, such as young kids and a growing career, that limit their availability for research or co-design activities, particularly if they are also managing health issues. In addition, measuring and evaluating the impact and effectiveness of patient involvement can be difficult, leading to questions about the value of their contributions. Gaining insight into patient involvement challenges in the development of information and services for adolescent and young adult cancer survivors could provide valuable guidance to HCPs to overcome these challenges.

### Lessons Learned

Collaboration between professionals, including online developers, researchers, and adolescents and young adults with lived experience, ensured a direct translation of insights into the platform. The involvement of professionals from various organizations in the project provided direct access to content creators for the platform, for instance, epidemiological research from the Integraal Kankercentrum Nederland (IKNL; Comprehensive Cancer Organisation) and technical expertise from Kanker.nl. An important aspect of working in a project group with various organizations is the discussion of mutual expectations and collaboration agreements before the start of the project. Project members working across different organizations were responsible for communicating project details and their collaborative design approach to their internal colleagues.

According to the project group, the involvement of adolescents and young adults with lived experience was considered very important in the development of an online platform. Several key elements appeared to be important in involving adolescents and young adults with lived experience: an inclusive and open atmosphere during the collaborative process, emphasizing the principles of equality and creating a welcoming environment. adolescents and young adults with lived experiences indicated that they wanted to be involved from the initial stage of the project in order to be able to contribute to the development of the project design. This also contributed to the feeling that the patient’s experience was important and valued, as well as equality and being a full partner in the project. Regular evaluation of the collaboration was essential to ensure continued alignment with the project among all members of the project group. It was valuable to involve different adolescents and young adults with lived experiences in the project in different ways and at different times during the development. In addition, the use of specialized terminology (jargon) could be a potential barrier to collaboration. Including a glossary of commonly used terms helped to bridge communication gaps, ensured that all the stakeholders spoke the same language, and facilitated a more inclusive and effective collaboration [50]. In light of these findings and also found in previous literature, such as the INVOLVE initiative from the United Kingdom [50], education for both patients and researchers could improve and guide collaboration between them. This should include an understanding of patient involvement, insights into the experiences of other patients and researchers regarding patient involvement (both facilitators and barriers), and practical skills for effective collaboration. But particularly when working in interorganizational settings, knowledge and understanding of each other’s activities and organizations could be helpful.

In this study, adolescents and young adults with lived experience reported that there was no formal financial compensation available for their contribution to the project group, but they did receive informal financial compensation in the form of gift cards. This was also reported in a recent systematic review by Grace Fox et al [51], where only 25% reported offering financial compensation, mostly in the form of honoraria or stipend, salary, or gift cards [52]. In this study, compensation for their contributions as adolescents and young adults with lived experiences was seen as a meaningful form of acknowledgment and demonstrated that their efforts were recognized and valued. They also mentioned the financial responsibilities they have, such as paying for a mortgage, rent, and supporting their family, all while coping with the consequences of their cancer diagnosis and treatment. Although there is previous research and guidelines on compensating patients for their contributions to health research [50,52,53], not all organizations and professionals are used to this and do not include these costs in the project budgets [52]. In line with our study, barriers hindering researchers from providing financial compensation were the limited funding and the lack of institutional guidance [52]. Organizations should have clear policies on their approach to compensating patients for their involvement, budgeting for patient involvement should be included in the research protocol and professionals should have guidance on this.

### Future Proof

Many developed interventions remain unused because they don’t make it out of the pilot phase due to limited funding. The COMPRAYA funding has provided the same start and leads to the opportunity to establish this “young and cancer” platform. However, given the temporary nature of this funding, it is important to consider its future beyond the funding period. An important aspect for choosing and creating the “young and cancer” platform within the existing Kanker.nl platform was its assurance of continued development and maintenance of the platform, as it is continuously funded by KWF. Formal agreements among stakeholders COMPRAYA, SJK, Dutch “Young and Cancer” Care Network and Kanker.nl ensure integration and further development of the “young and cancer” services in the future. The collaboration between COMPRAYA, SJK, and Kanker.nl is essential for ensuring continuous involvement in the platform. Within this agreement, the unique expertise of each organization is used. Kanker.nl plays a crucial role by hosting the platform on its website, leveraging its technical expertise in building and maintaining digital resources. Meanwhile, COMPRAYA, SJK, and Dutch adolescent and young adult “Young & Cancer” Care Network contribute valuable insights and input based on their specialized knowledge and experience on adolescent and young adult cancer survivors and their relatives based on scientific knowledge.

### Opportunities and Implications for the “Young and Cancer” Platform

As we continue to gain more insight into the (medical) care and support needs of adolescent and young adult cancer survivors and their relatives and how they might benefit from digital interventions, the information provision and services on the “young and cancer” platform need to be updated constantly. This includes exploring how to increase awareness of the online platform among adolescent and young adult cancer survivors. Insights from HCPs regarding their experiences with online services and the extent to which they inform adolescent and young adult survivors about this platform could also be helpful [54,55]. Conducting evaluation and assessing experiences and needs among visitors (both adolescent and young adult cancer survivors and their relatives) and HCPs will help identify what works well, what is lacking, and areas for improvement.

More research is needed to assess the use and impact of the “young and cancer” platform, focusing on the evolving needs and needs of underrepresented adolescent and young adult subgroups who might have different needs. For example, adolescents and young adults with an uncertain or poor prognosis [46], adolescents and young adults with low (health) literacy [46], adolescent and young adult cancer survivors with diverse cultural backgrounds, and Young Adult Childhood Cancer Survivors. More insight is needed into what already exists for these specific subgroups and to identify what is needed for customizing information and services to their specific needs. By continually refining the platform to meet adolescent and young adult cancer survivors’ needs, we aim to maximize its impact and utility.

### Limitations

The main limitation of this study was that there were no direct observations of the stakeholder consensus meetings and collaborative design meetings. Thereby, to evaluate the collaborative design, only project members were interviewed. Conducting interviews with a broader range of stakeholders with different backgrounds could enrich the findings and offer more interdisciplinary insights. The adolescents and young adults with lived experiences who were part of the project group were not representative of the adolescent and young adult population. They were all highly educated, and there was no diversity in terms of ethnicity. All had some experience of working as a patient representative, which can be seen as an advantage as they are familiar with the field and may have built up a network. However, the risk of having a “professional patient partner” is that their own patient experience may be sidelined and input become more similar to that of researchers. We have tried to minimize this by involving inexperienced adolescent and young adult cancer survivors at several points in time during the process, such as the stakeholder consensus meeting and by asking them to review the content of the platform.

### Conclusion

The collaborative design approach helped to integrate online information and services for adolescent and young adult cancer survivors. The collaboration between professionals, including online developers, researchers, and adolescents and young adults with lived experience, facilitated a direct translation of insights into the platform, while the support of the national cancer platform provides long-term sustainability. This resulted in the development of a dedicated sustainable “young and cancer” platform. This study highlights the importance of strategic stakeholder selection and intense involvement of the stakeholders through a collaborative design approach. Knowledge and understanding of each other’s activities and organizations can facilitate better communication and contribute to more integrated collaboration, especially when working on an interorganizational project.

## Appendix

Multimedia Appendix 1 COREQ checklist.

Multimedia Appendix 2 The various scenarios presented to stakeholders outlined different strategies for improving online information and services for adolescent and young adult cancer survivors, each varying in terms of investment and complexity.

Multimedia Appendix 3 Four personas representing adolescent and young adult cancer survivors and their relatives, covering various characteristics such as gender, tumor types, age, time since diagnosis, and platform needs.

Multimedia Appendix 4 A screenshot of the age-specific content on the "young and cancer" platform, related to fertility and pregnancy.

Multimedia Appendix 5 (Sub)themes of the evaluation of the collaboration within the project group.

## Abbreviations

COMPRAYA: COMPRehensive health outcome and intervention research among patients with adolescent and young adult cancer
COREQ: Consolidated Criteria for Reporting Qualitative Research
HCP: health care professional
IKNL: Integraal Kankercentrum Nederland
KWF: Koningin Wilhelmina Fonds
NFK: Nederlandse Federatie van Kankerpatiëntenorganisaties
SURVAYA: health-related quality of life and late effects among SURVivors of cancer in Adolescence and Young Adulthood
SJK: Stichting Jongeren en Kanker

## Data Availability

The datasets generated during and/or analyzed during this study are available from the corresponding author on reasonable request.
