# Supplementary material for: Developing and Integrating Digital Sources in an Accessible and Sustainable Online Platform for Adolescents and Young Adult Cancer Survivors: Collaborative Design Approach
Source: JMIR Form Res. 2025 Jul 11;9:e60897. doi: 10.2196/60897 (PMC12299946; doi:10.2196/60897)
Supplement: Multimedia Appendix 1 [file formative_v9i1e60897_app1.docx]

**Consolidated criteria for reporting qualitative studies (COREQ): 32-item checklist**

| **Topic** | **Item No.** | **Guide questions/description** | **Reported on Page #** |
| --- | --- | --- | --- |
| **Domain 1: Research team and reﬂexivity** |  |  |  |
| *Personal Characteristics* |  |  |  |
| Inter viewer/facilitator | 1 | Which author/s conducted the interview or focus group? | Page 8 |
| Credentials | 2 | What were the researcher’s credentials? E.g. PhD, MD | Page 8 |
| Occupation | 3 | What was their occupation at the time of the study? | Page 8 |
| Gender | 4 | Was the researcher male or female? | Page 8 |
| Experience and training | 5 | What experience or training did the researcher have? | Page 8 |
| *Relationship with participants* |  |  |  |
| Relationship established | 6 | Was a relationship established prior to study commencement? | No |
| Participant knowledge of the interviewer | 7 | What did the participants know about the researcher? e.g. personal goals, reasons for doing the research | Page 8 |
| Interviewer characteristics | 8 | What characteristics were reported about the interviewer/facilitator? e.g. Bias, assumptions, reasons and interests in the research topic | Page 8 |
| **Domain 2: study design** |  |  |  |
| *Theoretical framework* |  |  |  |
| Methodological orientation and Theory | 9 | What methodological orientation was stated to underpin the study? e.g. grounded theory, discourse analysis, ethnography, phenomenology, content analysis | Page 9 |
| *Participant selection* |  |  |  |
| Sampling | 10 | How were participants selected? e.g. purposive, convenience, consecutive, snowball | Page 8 |
| Method of approach | 11 | How were participants approached? e.g. face-to-face, telephone, mail, email | Page 8 |
| Sample size | 12 | How many participants were in the study? | Table 3 |
| Non-participation | 13 | How many people refused to participate or dropped out? Reasons? | Page 16 |
| *Setting* |  |  |  |
| Setting of data collection | 14 | Where was the data collected? e.g. home, clinic, workplace | Page 8 |
| Presence of non-participants | 15 | Was anyone else present besides the participants and researchers? | No |
| Description of sample | 16 | What are the important characteristics of the sample? e.g. demographic data, date | Table 3 |
| *Data collection* |  |  |  |
| Interview guide | 17 | Were questions, prompts, guides provided by the authors? Was it pilot tested? | No |
| Repeat interviews | 18 | Were repeat interviews carried out? If yes, how many? | No |
| Audio/visual recording | 19 | Did the research use audio or visual recording to collect the data? | Page 8 |
| Field notes | 20 | Were ﬁeld notes made during and/or after the interview or focus group? | No |
| Duration | 21 | What was the duration of the interviews or focus group? | Page 8 |
| Data saturation | 22 | Was data saturation discussed? | No |
| Transcripts returned | 23 | Were transcripts returned to participants for comment and/or correction? | No |
| **Domain 3: analysis and ﬁndings** |  |  |  |
| *Data analysis* |  |  |  |
| Number of data coders | 24 | How many data coders coded the data? | Page 9 |
| Description of the coding tree | 25 | Did authors provide a description of the coding tree? | No |
| Derivation of themes | 26 | Were themes identiﬁed in advance or derived from the data? | Derived from data |
| Software | 27 | What software, if applicable, was used to manage the data? | NVivo |
| Participant checking | 28 | Did participants provide feedback on the ﬁndings? | Discussed with SS (project group member) |
| *Reporting* |  |  |  |
| Quotations presented | 29 | Were participant quotations presented to illustrate the themes/ﬁndings? Was each quotation identiﬁed? e.g. participant number | Appendix 5 |
| Data and ﬁndings consistent | 30 | Was there consistency between the data presented and the ﬁndings? | Yes |
| Clarity of major themes | 31 | Were major themes clearly presented in the ﬁndings? | Yes |
| Clarity of minor themes | 32 | Is there a description of diverse cases or discussion of minor themes? | Yes |
