# Supplementary material for: Developing and Integrating Digital Sources in an Accessible and Sustainable Online Platform for Adolescents and Young Adult Cancer Survivors: Collaborative Design Approach
Source: JMIR Form Res. 2025 Jul 11;9:e60897. doi: 10.2196/60897 (PMC12299946; doi:10.2196/60897)
Supplement: Multimedia Appendix 2 [file formative_v9i1e60897_app2.pptx]

## Slide 1
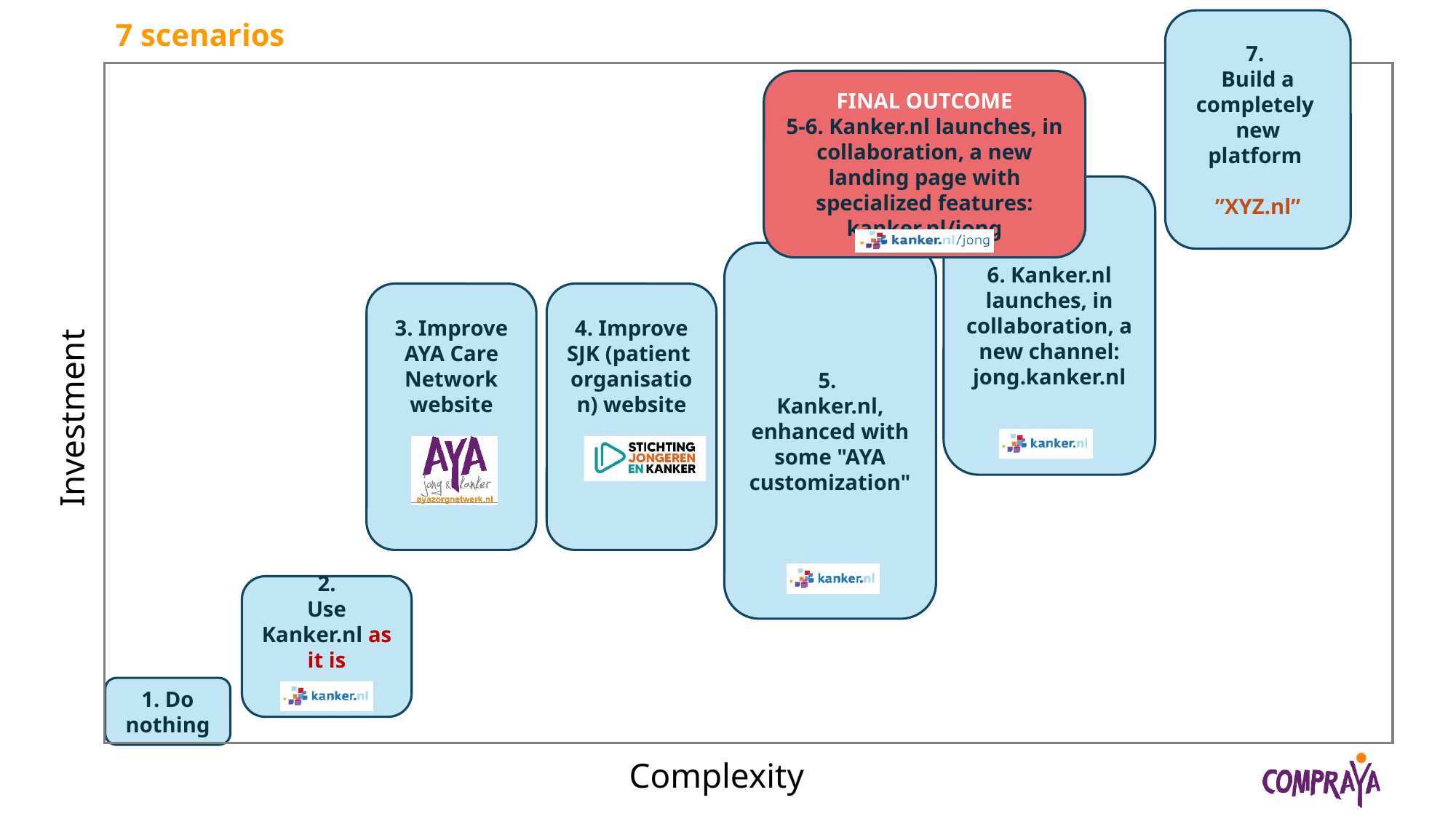

# 7 scenarios
7.
Build a completely 
new platform 
”XYZ.nl”
FINAL OUTCOME
5-6. Kanker.nl launches, in collaboration, a new landing page with specialized features: kanker.nl/jong
6. Kanker.nl launches, in collaboration, a new channel: jong.kanker.nl
5.
Kanker.nl, enhanced with some "AYA customization"
3. Improve AYA Care Network website
4. Improve SJK (patient 
organisation) website
Investment
2.
Use Kanker.nl as it is
1. Do nothing
Complexity
