# Supplementary material for: Developing and Integrating Digital Sources in an Accessible and Sustainable Online Platform for Adolescents and Young Adult Cancer Survivors: Collaborative Design Approach
Source: JMIR Form Res. 2025 Jul 11;9:e60897. doi: 10.2196/60897 (PMC12299946; doi:10.2196/60897)
Supplement: Multimedia Appendix 3 [file formative_v9i1e60897_app3.pptx]

## Slide 1
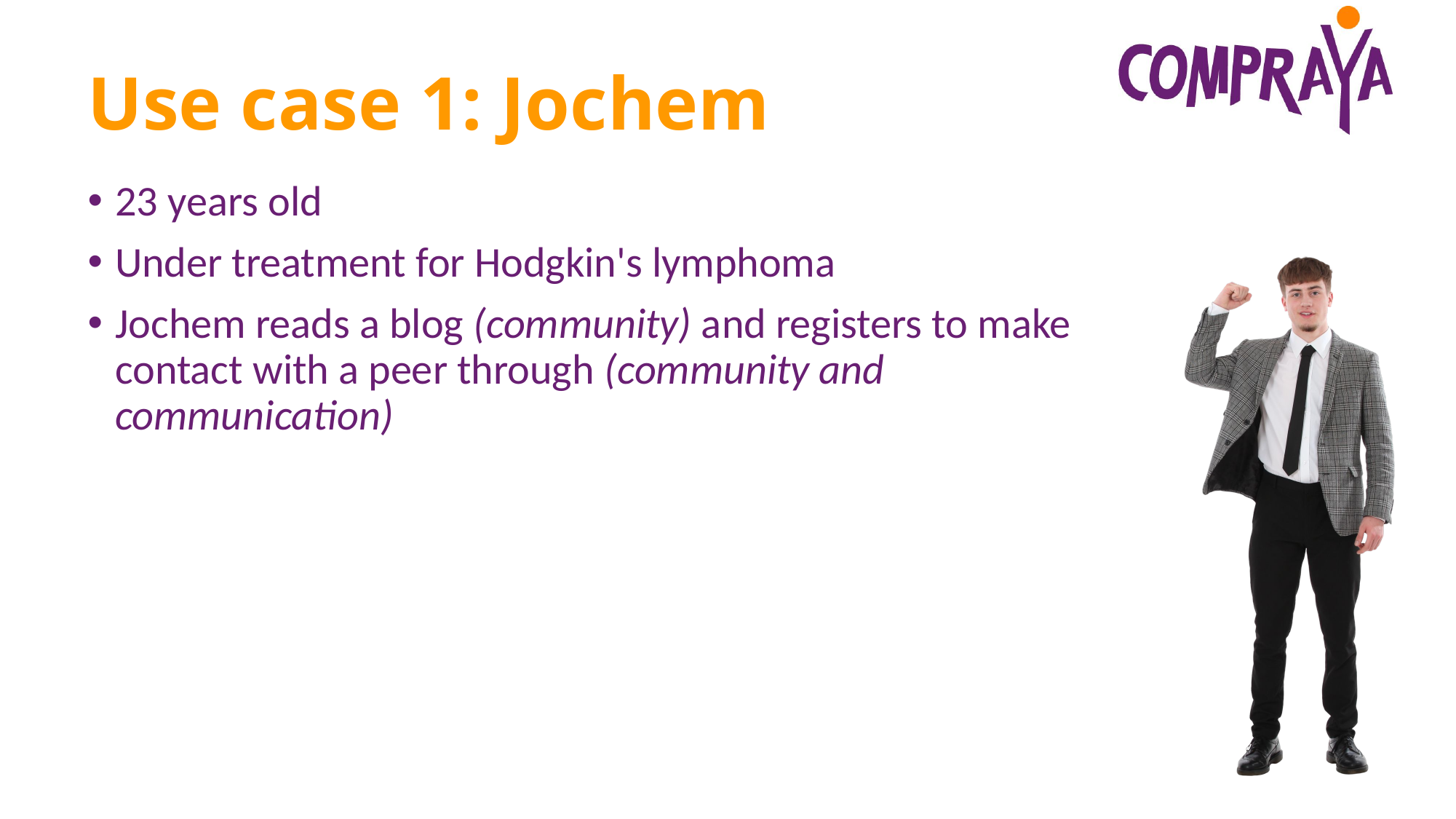

# Use case 1: Jochem
23 years old
Under treatment for Hodgkin's lymphoma
Jochem reads a blog (community) and registers to make contact with a peer through (community and communication)

## Slide 2
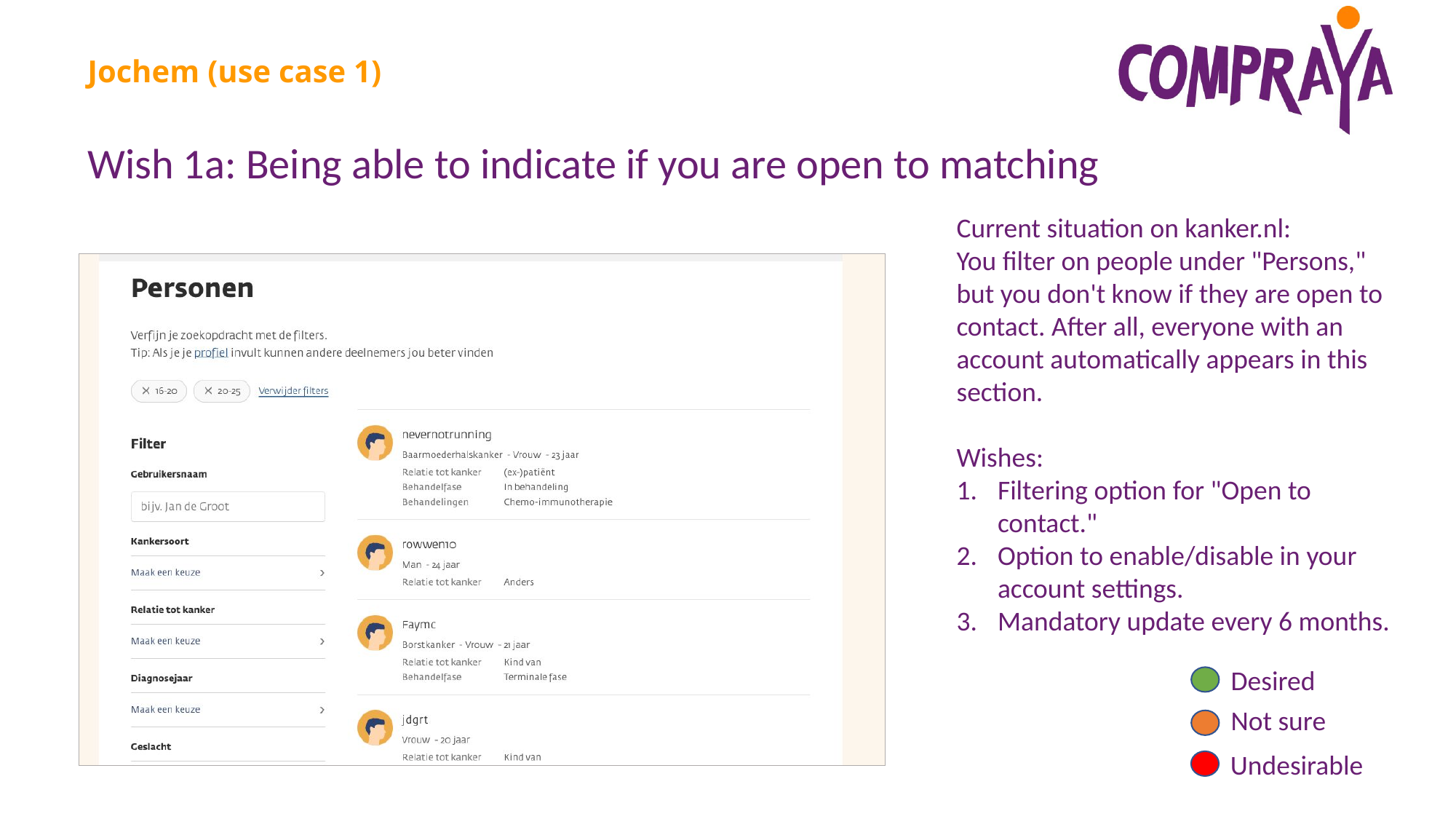

Jochem (use case 1)
Wish 1a: Being able to indicate if you are open to matching
Current situation on kanker.nl:
You filter on people under "Persons," but you don't know if they are open to contact. After all, everyone with an account automatically appears in this section.
Wishes:
Filtering option for "Open to contact."
Option to enable/disable in your account settings.
Mandatory update every 6 months.
Desired
Not sure
Undesirable

## Slide 3
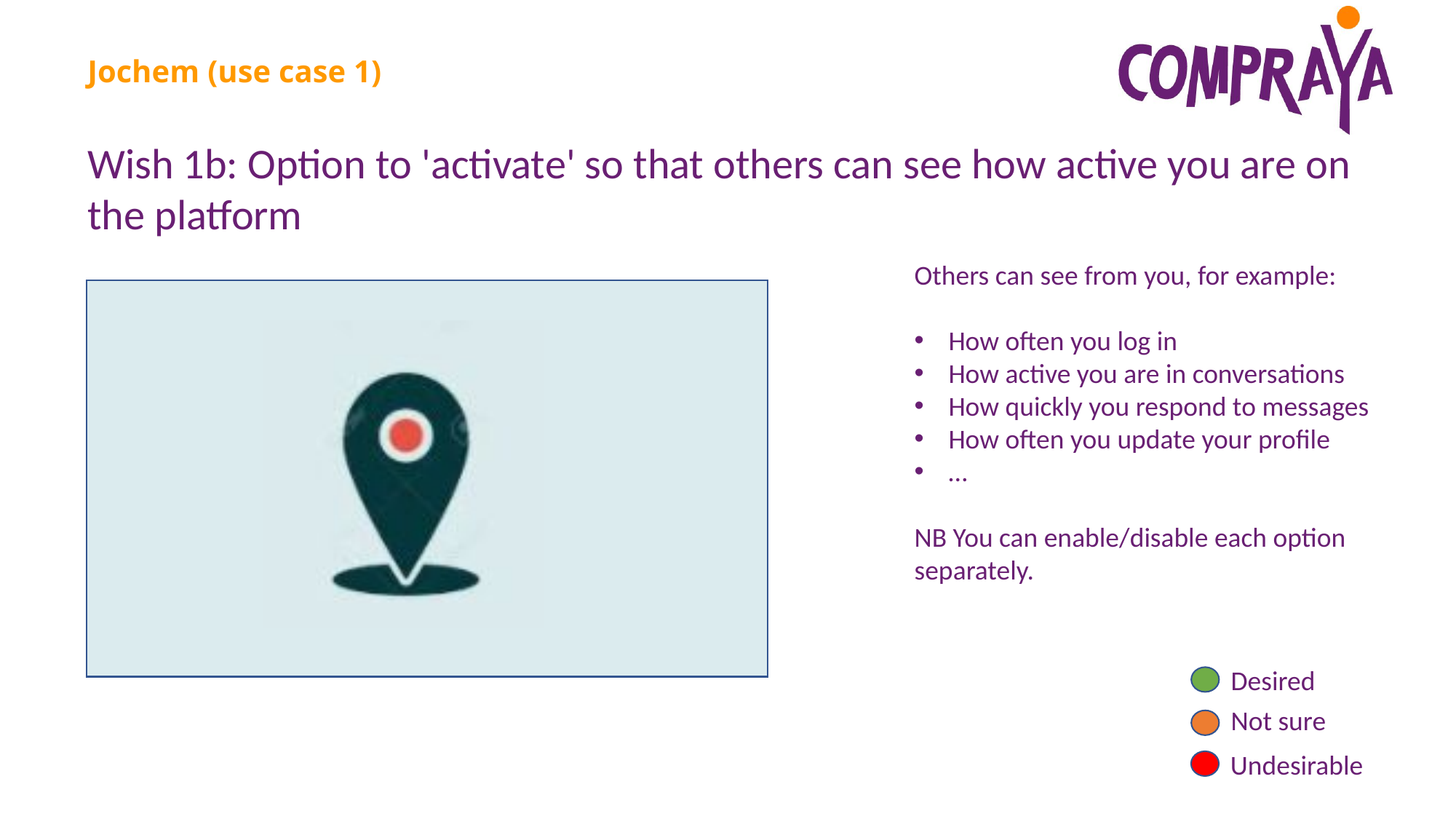

Jochem (use case 1)
Wish 1b: Option to 'activate' so that others can see how active you are on the platform
Others can see from you, for example:
How often you log in
How active you are in conversations
How quickly you respond to messages
How often you update your profile
…
NB You can enable/disable each option separately.
Desired
Not sure
Undesirable

## Slide 4
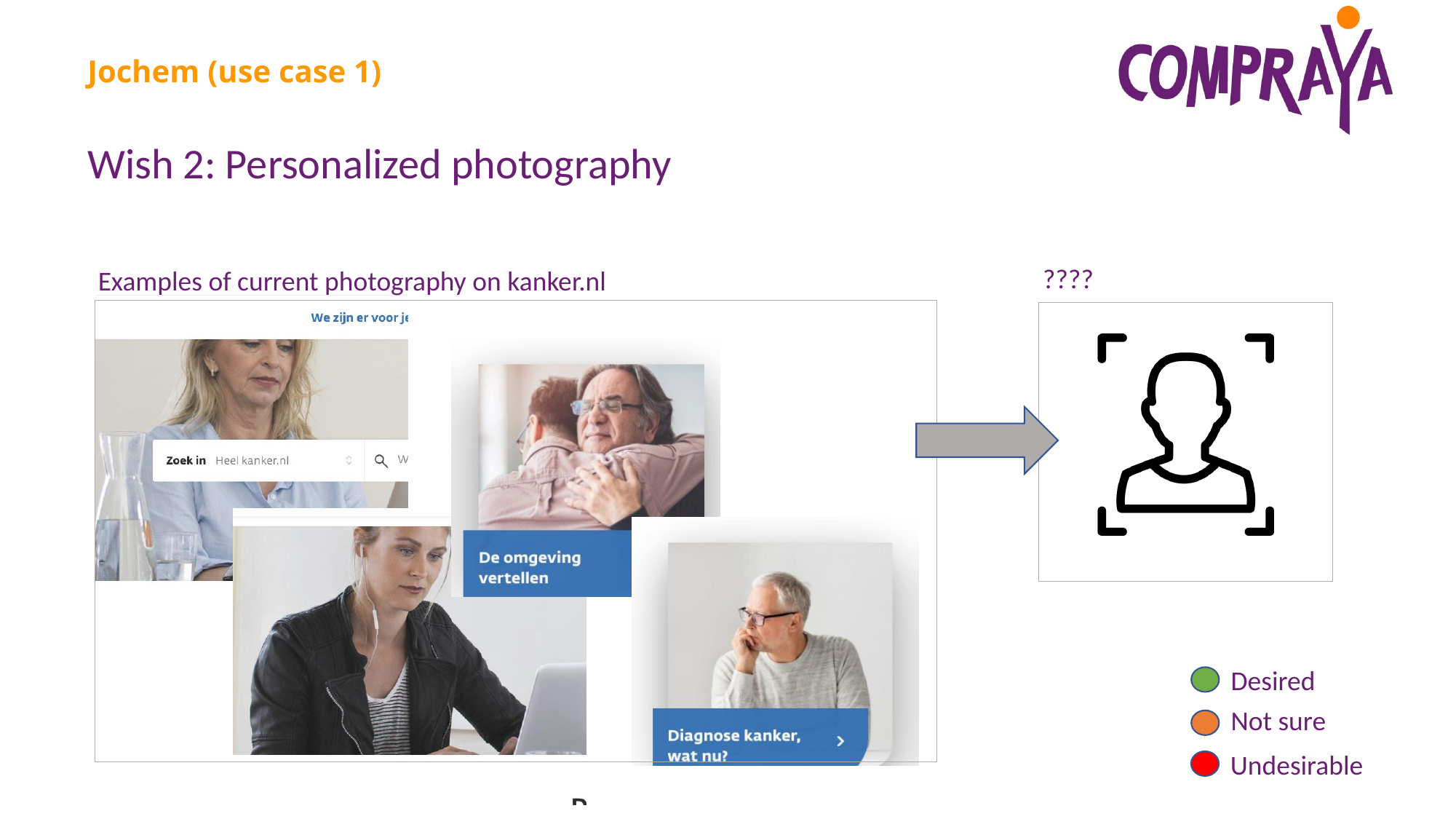

Jochem (use case 1)
Wish 2: Personalized photography
????
Examples of current photography on kanker.nl
Desired
Not sure
Undesirable

## Slide 5
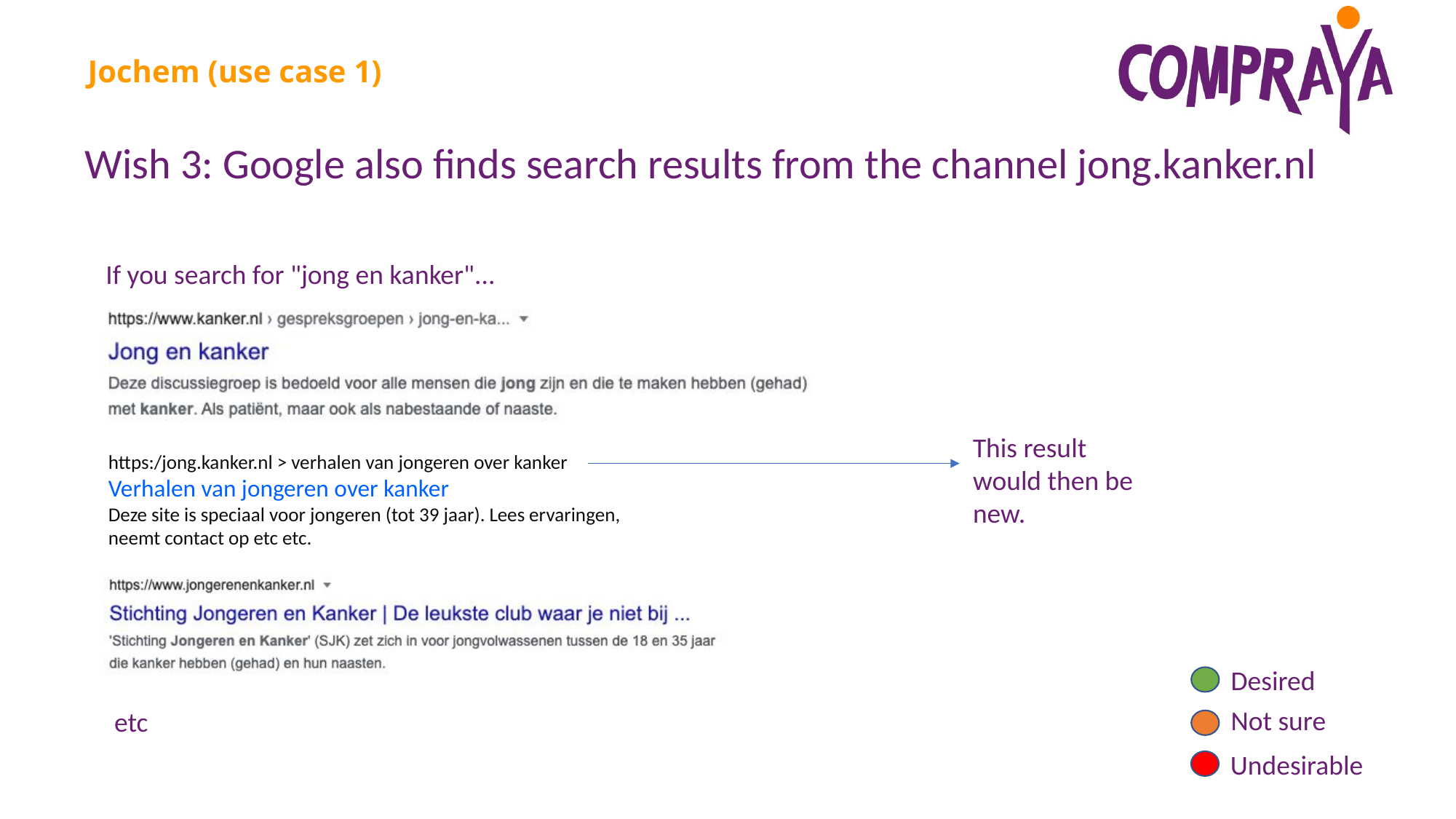

# Jochem (use case 1)
Wish 3: Google also finds search results from the channel jong.kanker.nl
If you search for "jong en kanker"...
This result would then be new.
https:/jong.kanker.nl > verhalen van jongeren over kanker
Verhalen van jongeren over kanker
Deze site is speciaal voor jongeren (tot 39 jaar). Lees ervaringen, neemt contact op etc etc.
Desired
Not sure
etc
Undesirable

## Slide 6
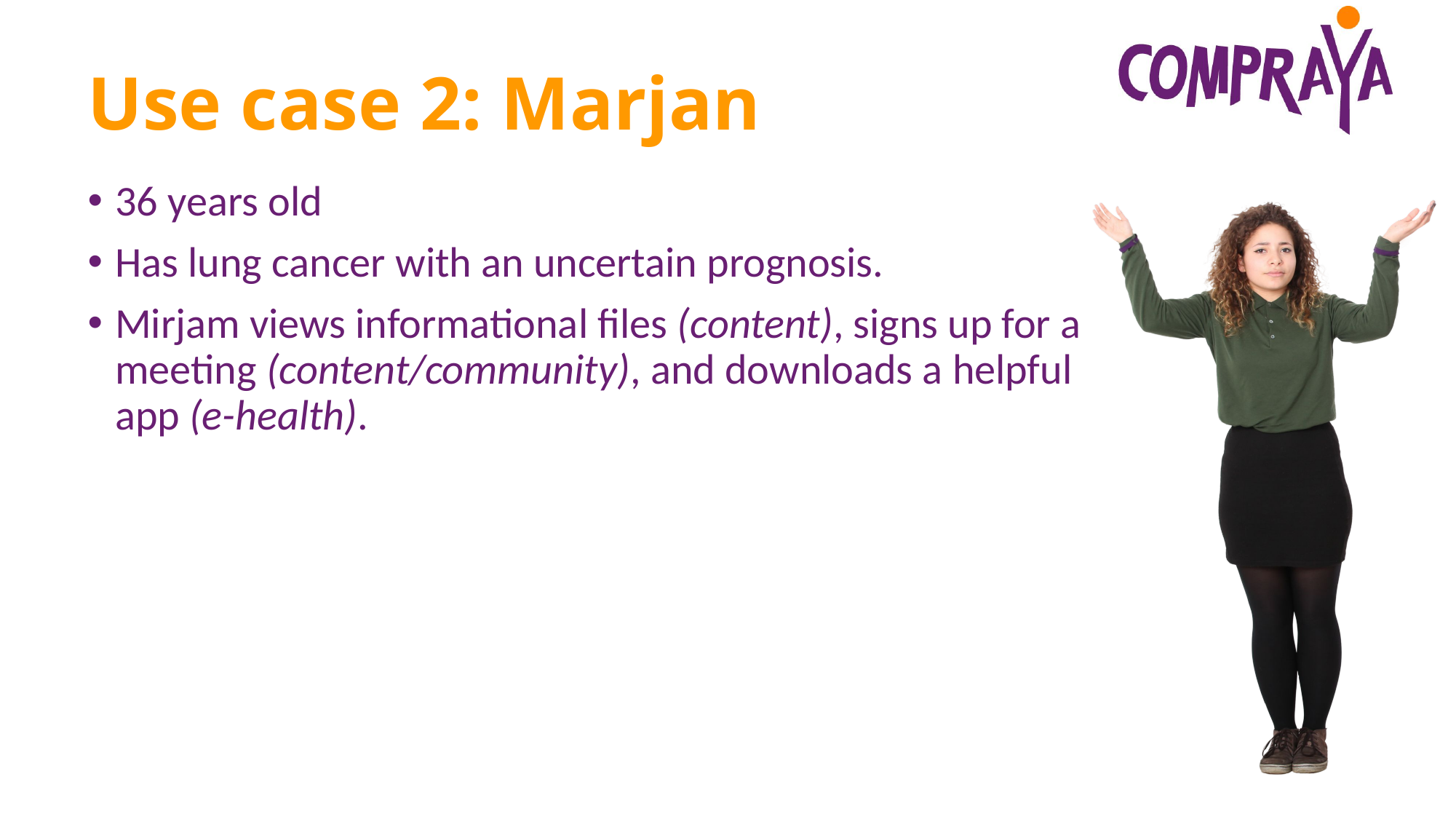

# Use case 2: Marjan
36 years old
Has lung cancer with an uncertain prognosis.
Mirjam views informational files (content), signs up for a meeting (content/community), and downloads a helpful app (e-health).

## Slide 7
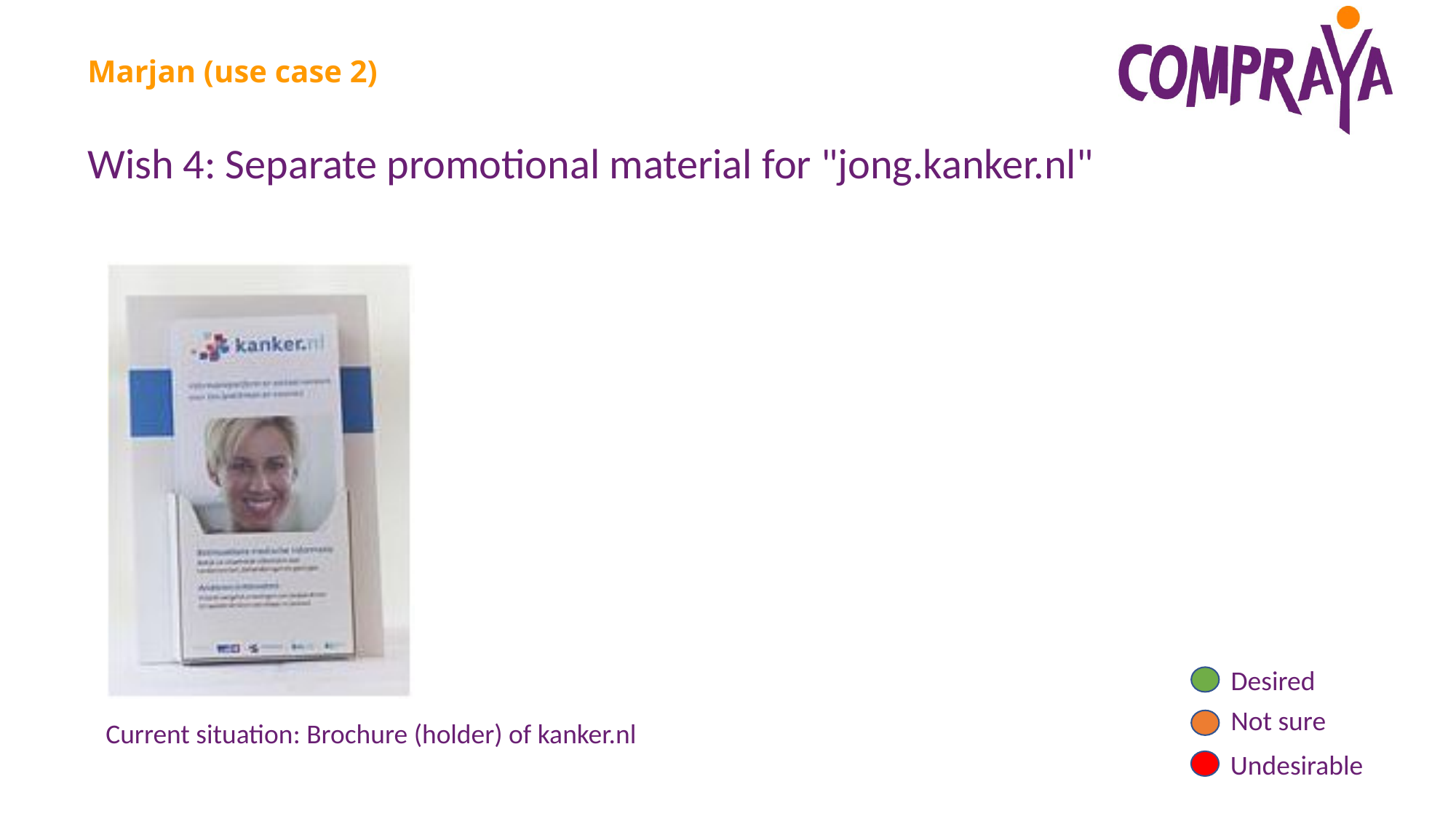

Marjan (use case 2)
Wish 4: Separate promotional material for "jong.kanker.nl"
Desired
Not sure
Current situation: Brochure (holder) of kanker.nl
Undesirable

## Slide 8
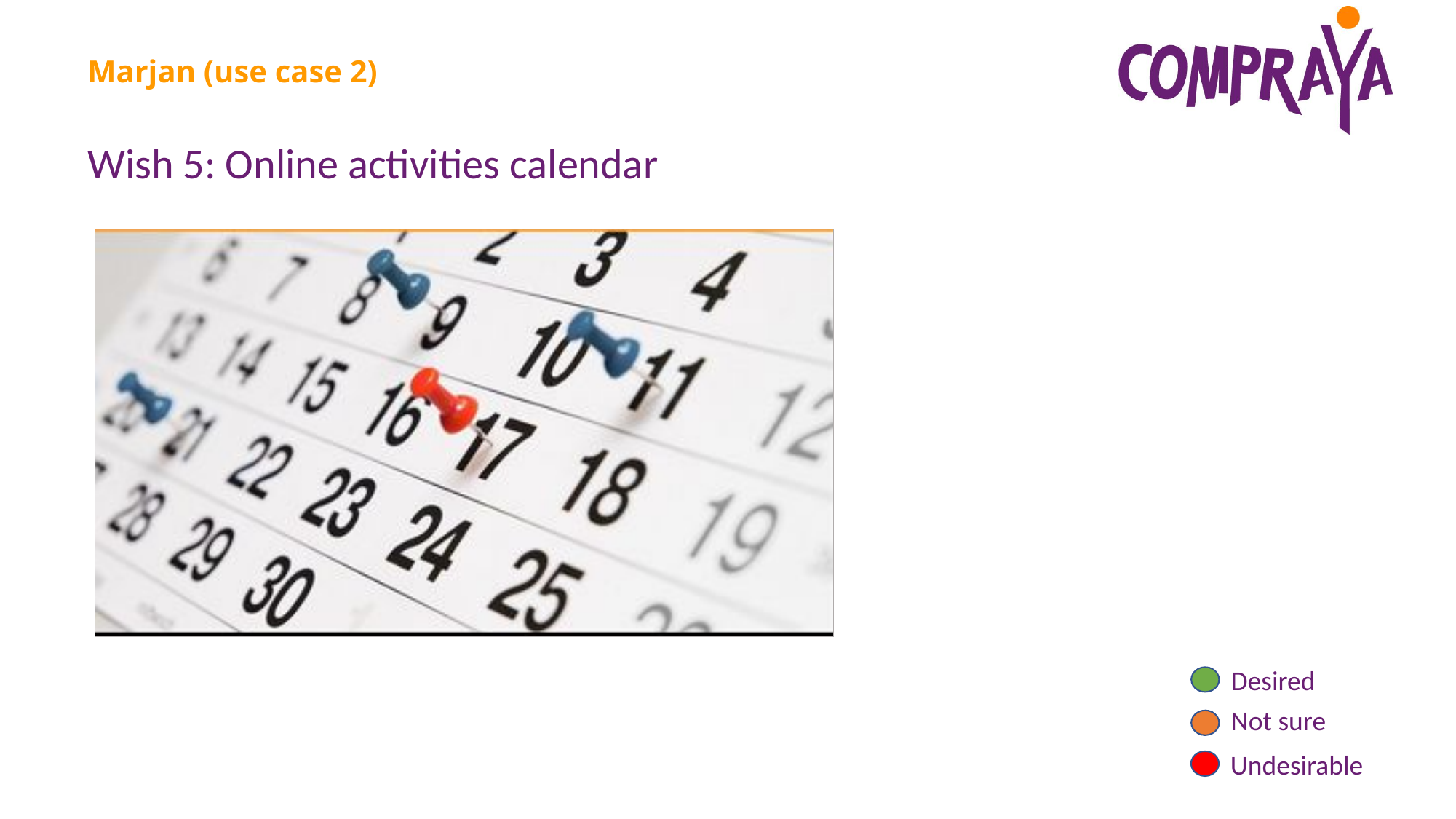

Marjan (use case 2)
Wish 5: Online activities calendar
Desired
Not sure
Undesirable

## Slide 9
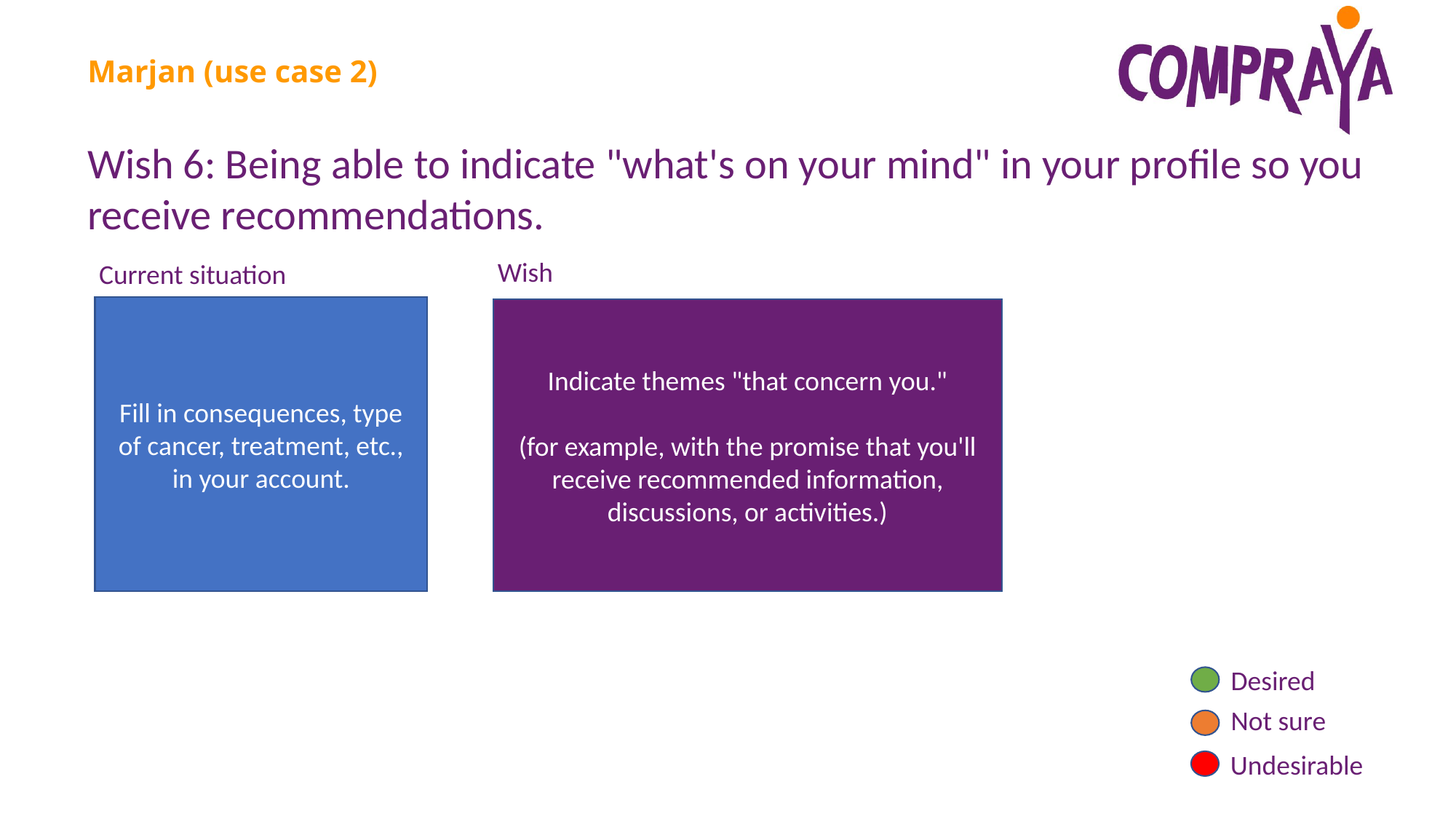

Marjan (use case 2)
Wish 6: Being able to indicate "what's on your mind" in your profile so you receive recommendations.
Wish
Current situation
Fill in consequences, type of cancer, treatment, etc., in your account.
Indicate themes "that concern you."
(for example, with the promise that you'll receive recommended information, discussions, or activities.)
Desired
Not sure
Undesirable

## Slide 10
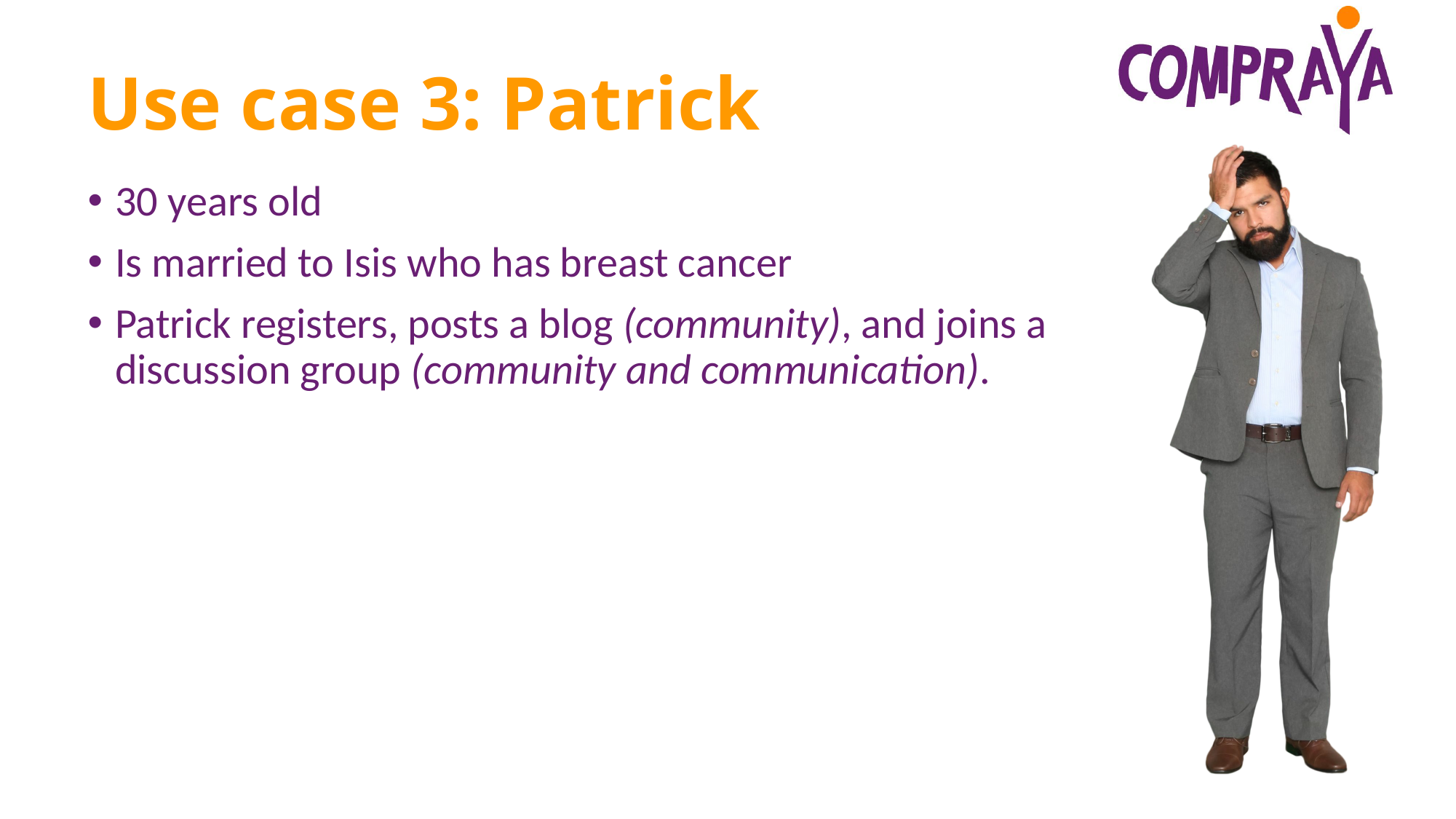

# Use case 3: Patrick
30 years old
Is married to Isis who has breast cancer
Patrick registers, posts a blog (community), and joins a discussion group (community and communication).

## Slide 11
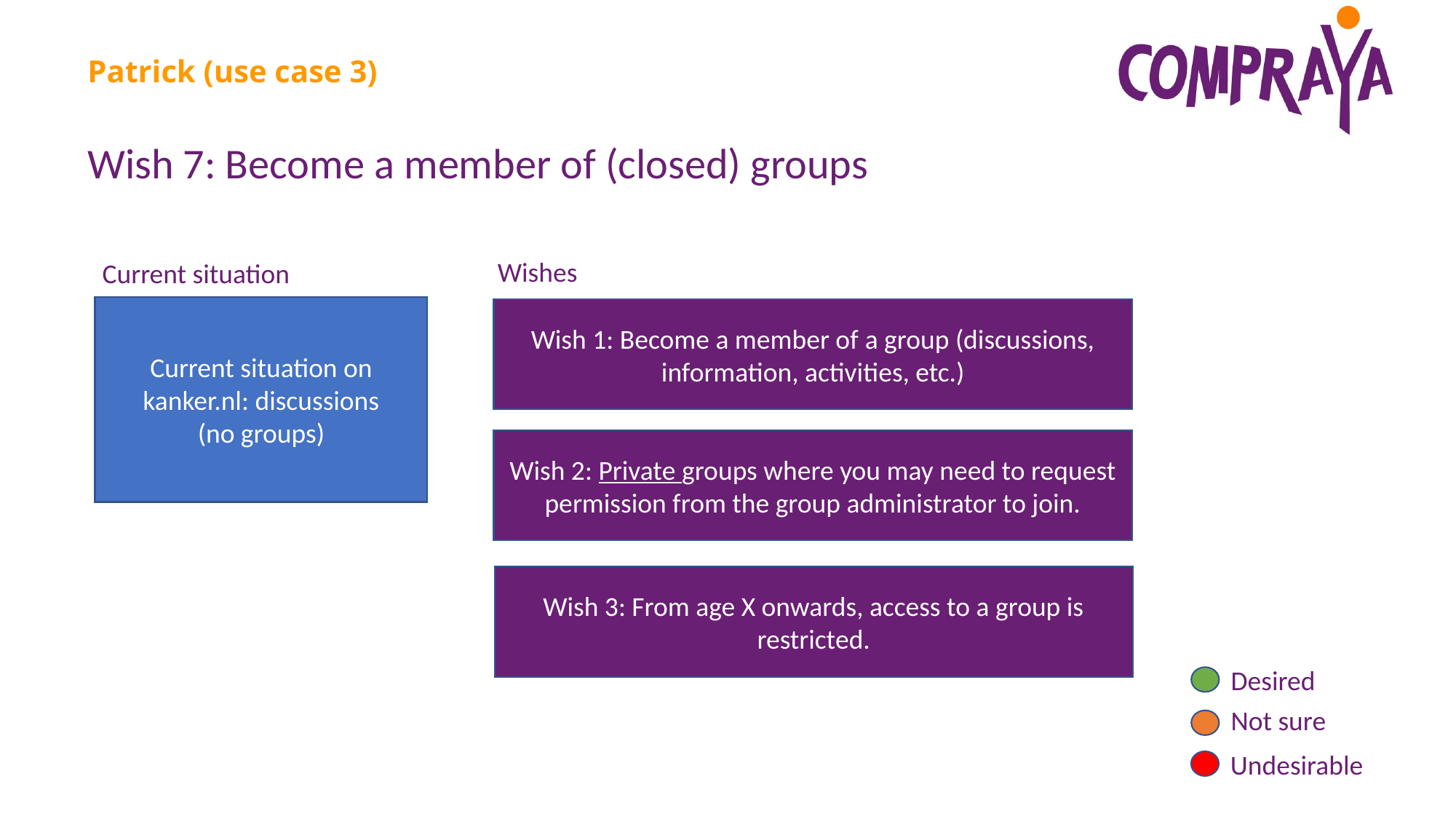

Patrick (use case 3)
Wish 7: Become a member of (closed) groups
Wishes
Current situation
Current situation on kanker.nl: discussions
(no groups)
Wish 1: Become a member of a group (discussions, information, activities, etc.)
Wish 2: Private groups where you may need to request permission from the group administrator to join.
Wish 3: From age X onwards, access to a group is restricted.
Desired
Not sure
Undesirable

## Slide 12
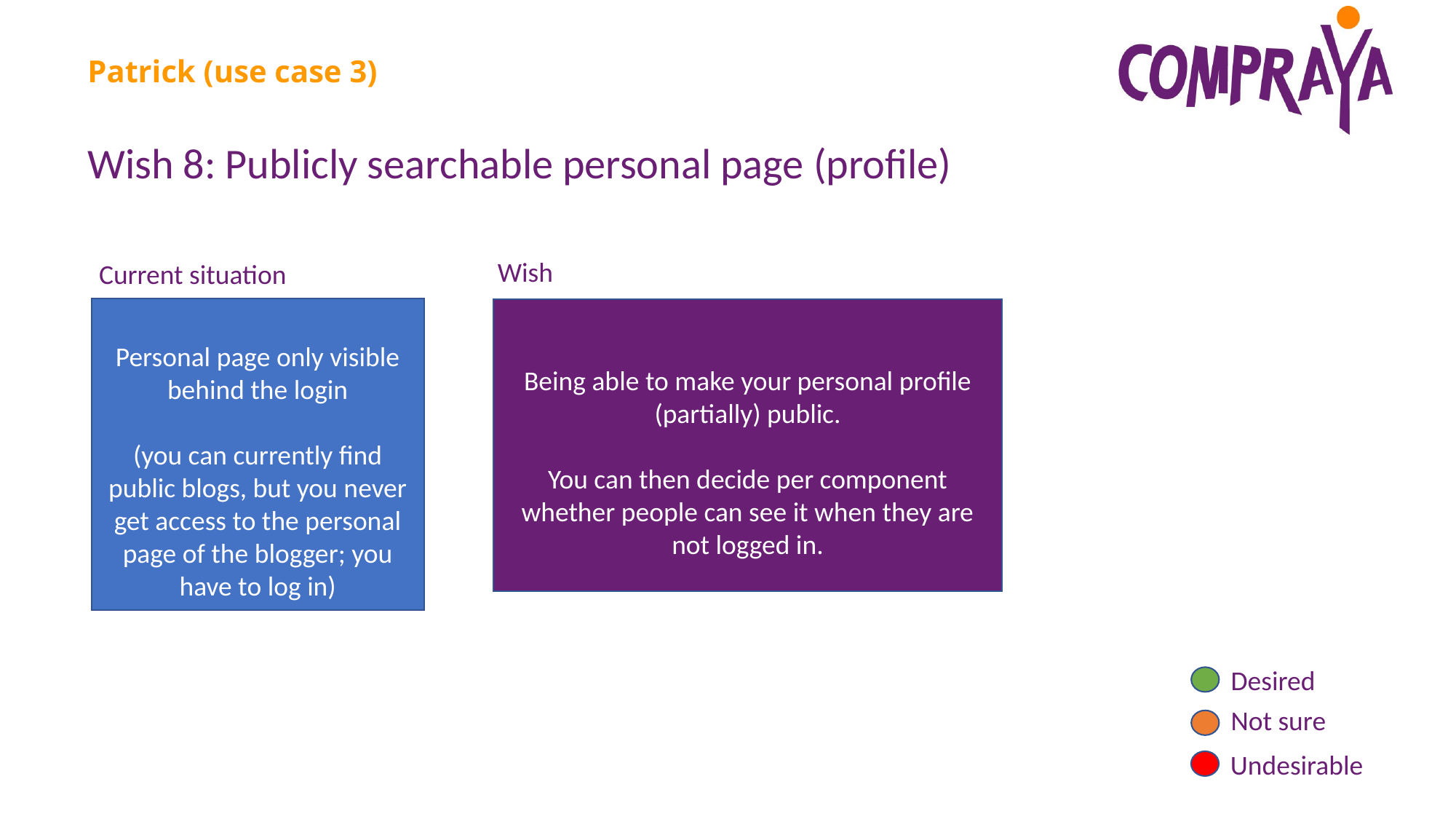

Patrick (use case 3)
Wish 8: Publicly searchable personal page (profile)
Wish
Current situation
Personal page only visible behind the login
(you can currently find public blogs, but you never get access to the personal page of the blogger; you have to log in)
Being able to make your personal profile (partially) public.
You can then decide per component whether people can see it when they are not logged in.
Desired
Not sure
Undesirable

## Slide 13
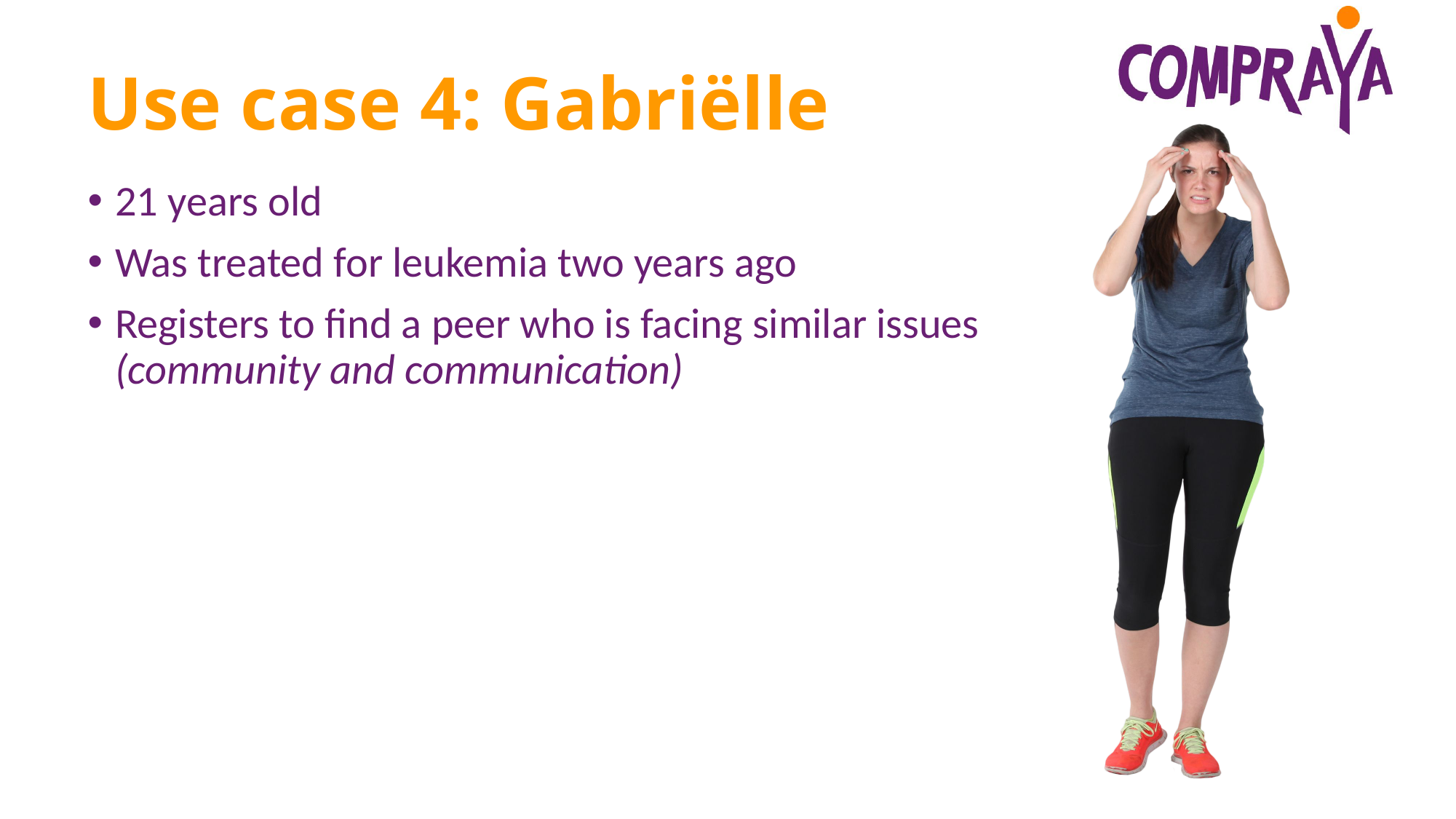

# Use case 4: Gabriëlle
21 years old
Was treated for leukemia two years ago
Registers to find a peer who is facing similar issues (community and communication)

## Slide 14
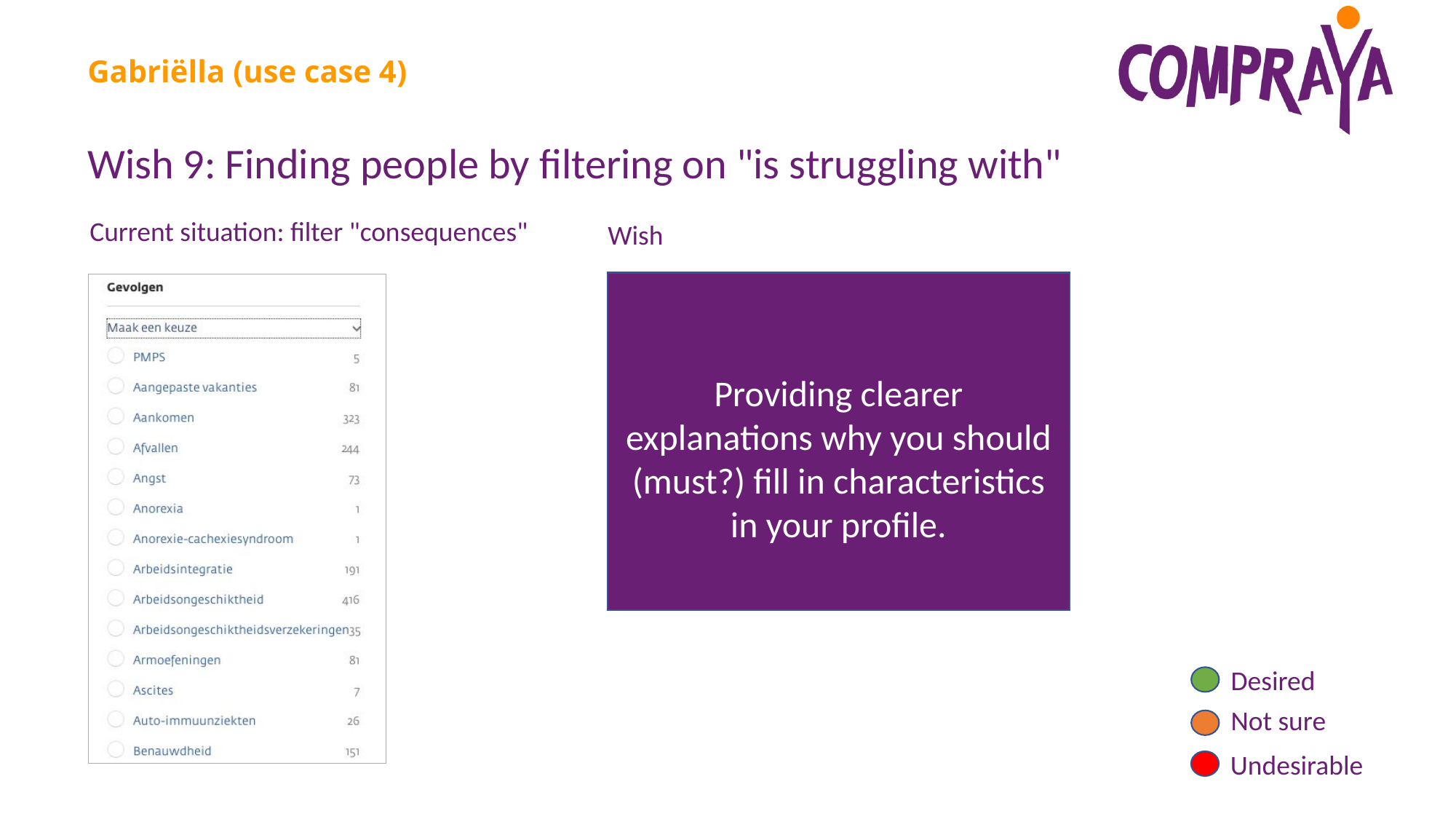

Gabriëlla (use case 4)
Wish 9: Finding people by filtering on "is struggling with"
Current situation: filter "consequences"
Wish
Providing clearer explanations why you should (must?) fill in characteristics in your profile.
Desired
Not sure
Undesirable

## Slide 15
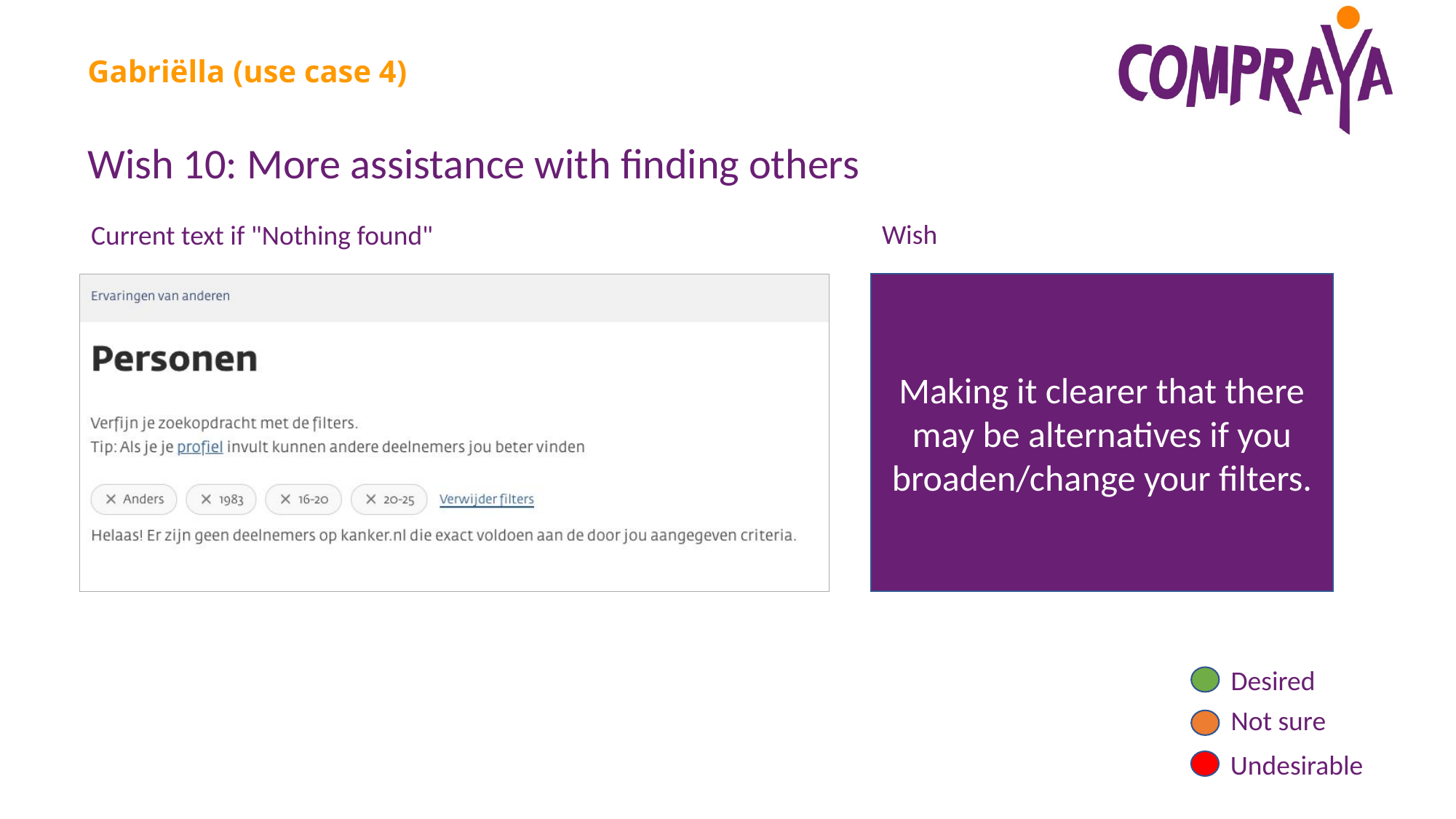

Gabriëlla (use case 4)
Wish 10: More assistance with finding others
Wish
Current text if "Nothing found"
Making it clearer that there may be alternatives if you broaden/change your filters.
Desired
Not sure
Undesirable
