# Supplementary figures and images for: Developing and Integrating Digital Sources in an Accessible and Sustainable Online Platform for Adolescents and Young Adult Cancer Survivors: Collaborative Design Approach
Source: JMIR Form Res. 2025 Jul 11;9:e60897. doi: 10.2196/60897 (PMC12299946; doi:10.2196/60897)

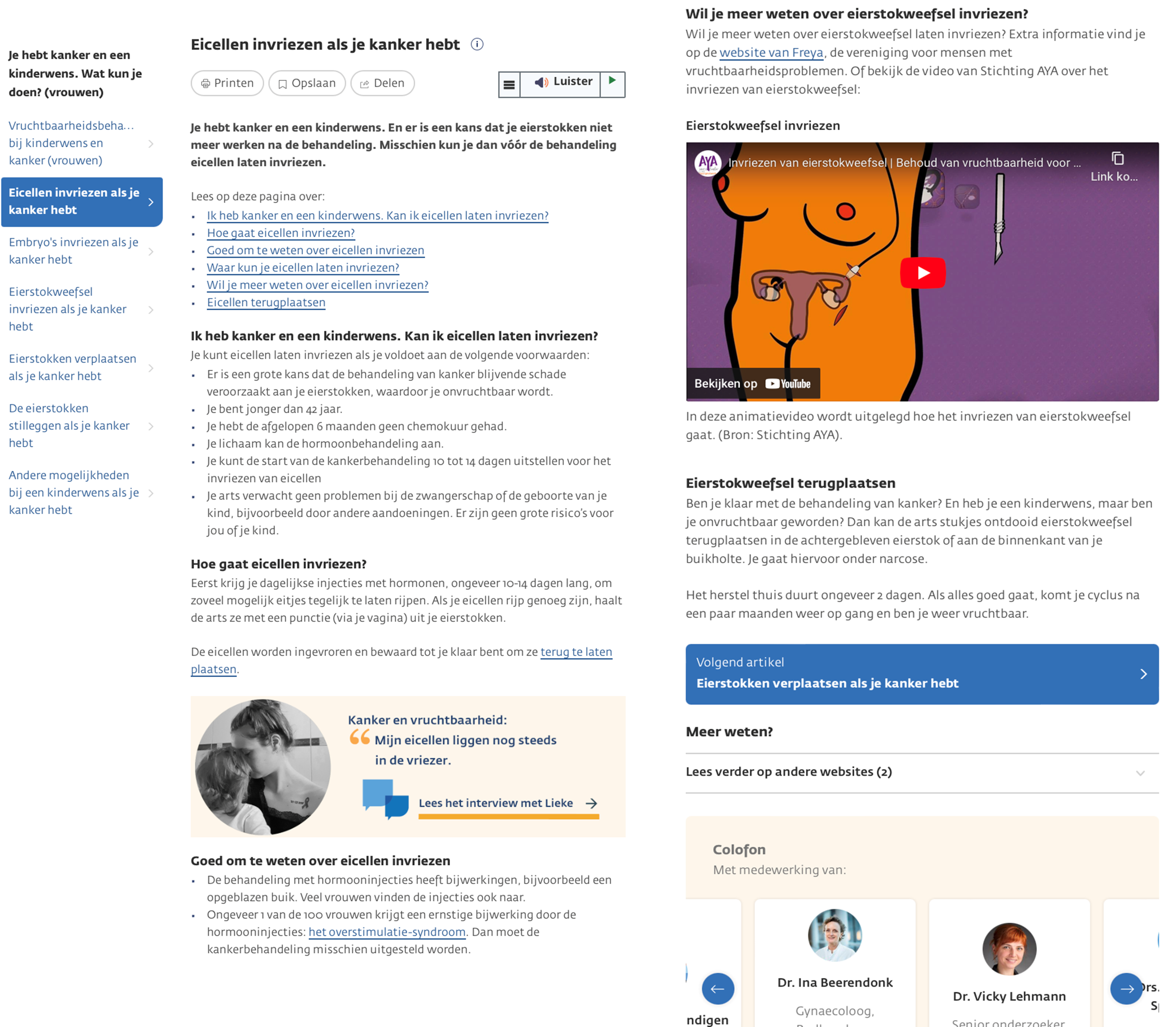

Supplement: Multimedia Appendix 4 [file formative_v9i1e60897_app4.png]
